# Epigenetic Age Acceleration as a Modifiable Public Health Target: A Systematic Review and Meta-Analysis of Environmental, Behavioral, and Social Determinants with Development of the MEAB-Index

**DOI:** 10.3390/ijms27115032

**Published:** 2026-06-02

**Authors:** Silvana Mirella Aliberti, Piergiorgio Marigliano, Mario Capunzo

**Affiliations:** 1 Department of Medicine, Surgery and Dentistry “Scuola Medica Salernitana”, University of Salerno, 84081 Salerno, Italy; mcapunzo@unisa.it; 2 Division of Preventive Medicine, Dresden International University (DIU), 01067 Dresden, Germany; 3 Department of Economics, eCampus University, 22060 Como, Italy; piergiorgio.marigliano@uniecampus.it; 4 Complex Operational Unit of Health and Hygiene, University Hospital “San Giovanni di Dio e Ruggi d’Aragona”, 84131 Salerno, Italy

**Keywords:** epigenetic age acceleration, DNA methylation clocks, modifiable determinants, systematic review, meta-analysis, preventable aging, MEAB-Index, public health

## Abstract

Chronological age is a poor indicator of interindividual differences in biological aging. DNA methylation-based epigenetic clocks provide a reliable measure of biological age and epigenetic age acceleration (EAA). Although modifiable behavioral, environmental, and social factors appear to influence EAA, the magnitude, consistency, and potential preventability of these associations have never been systematically quantified. We conducted a systematic review and meta-analysis following PRISMA 2020 guidelines. PubMed/MEDLINE and Scopus were searched from inception to 7 April 2026 for English-language observational and interventional studies reporting quantitative associations between modifiable determinants and EAA measured using validated clocks (Horvath, PhenoAge, GrimAge, DunedinPACE). Effect sizes were harmonized into four analytical pools. Random-effects meta-analyses were performed using the DerSimonian–Laird estimator, with pre-specified subgroup analyses by exposure category. Heterogeneity, publication bias, and robustness were thoroughly assessed. A novel Modifiable Epigenetic Aging Burden Index (MEAB-Index) was developed to quantify the cumulative preventable burden. Only studies conducted in adult populations (≥18 years) were eligible. Eighty-three studies providing 118 distinct exposure–clock associations were included. In the primary analysis (Pool A, *n* = 60), adverse modifiable exposures were associated with accelerated EAA (pooled β = +0.310 years per unit exposure, 95% CI 0.255–0.366). The strongest associations were observed for metabolic and inflammatory markers (β = +0.913) and environmental exposures (β = +0.466). The MEAB-Index yielded a Cumulative Preventable Burden of +1.566 years (bootstrap 95% CI 1.011–2.123). Findings were robust across sensitivity analyses and remained directionally consistent in secondary pools (B–D). This study provides the most comprehensive quantitative synthesis to date on the modifiability of epigenetic aging. Our findings demonstrate that EAA is meaningfully shaped by behavioral, environmental, and social determinants. The MEAB-Index introduces a novel framework for estimating the preventable burden of biological aging and for prioritizing interventions. Reducing key modifiable risk factors, particularly metabolic/inflammatory and environmental exposures, could substantially slow biological aging at the population level and support the transition toward ageing-centered preventive strategies.

## 1. Introduction

Population aging represents one of the greatest public health challenges of the 21st century. According to United Nations projections, by 2050 the global population aged ≥65 years will exceed 1.6 billion, with a substantial increase also in the number of centenarians [1]. While chronological age remains the most widely used metric to describe aging, it captures only a limited proportion of the marked interindividual heterogeneity in aging trajectories [2]. Individuals of the same chronological age may exhibit profoundly different functional, physiological, and clinical profiles, ranging from accelerated decline to preserved health and resilience at advanced ages. This variability has driven the field of geroscience to identify robust biomarkers capable of capturing biological aging processes more accurately than chronological age alone [3].

Over the past decade, DNA methylation-based epigenetic clocks have emerged as the most reliable and reproducible biomarkers of biological aging. The first-generation multi-tissue clock developed by Horvath [4] demonstrated high accuracy in predicting chronological age using a set of CpG sites selected through machine learning approaches. Subsequently, second-generation clocks, such as PhenoAge [5] and GrimAge [6], were designed to incorporate clinical and mortality-related phenotypes, thereby improving their predictive validity for health outcomes. More recently, third-generation clocks, including DunedinPACE [7], have shifted focus toward quantifying the dynamic pace of aging. Collectively, these measures exhibit strong associations with clinically relevant endpoints, including cardiovascular disease, metabolic disorders, frailty, and all-cause mortality, supporting their role as integrative biomarkers of systemic aging processes [8,9,10].

Epigenetic age acceleration (EAA), typically defined as the residual of epigenetic age regressed on chronological age, has been increasingly recognized not only as a predictor of adverse health outcomes but also as a potentially modifiable phenotype [11]. In this review, we focus exclusively on adult populations (≥18 years), as epigenetic aging trajectories and their modifiable determinants differ substantially between developmental and adult life stages. A growing body of evidence suggests that behavioral, environmental, and social determinants play a substantial role in shaping EAA [12]. Adverse exposures—including smoking, physical inactivity, air pollution, chronic psychosocial stress, and socioeconomic disadvantage—have been consistently associated with accelerated epigenetic age, whereas protective factors such as regular physical activity, adherence to healthy dietary patterns (e.g., Mediterranean diet), smoking cessation, and strong social support appear to mitigate or even reverse EAA [12,13,14]. These findings raise the possibility that biological aging may be, at least in part, preventable through modifiable exposures.

Despite the rapid expansion of the literature, the evidence base remains fragmented and largely descriptive. Existing systematic reviews have primarily focused on specific exposure domains or on the prognostic value of EAA. For example, Ryan et al. [12] summarized lifestyle and environmental correlates of epigenetic age, while Oblak et al. [9] examined social and environmental determinants. More recent work has addressed specific domains such as socioeconomic inequalities [15] or cardiometabolic conditions [16]. Although some meta-analyses have evaluated the association between EAA and health outcomes, no study has systematically quantified and harmonized the effects of major modifiable behavioral, environmental, and social determinants on EAA using a unified analytical framework. The absence of such a synthesis limits comparability across studies and hinders the translation of findings into actionable public health strategies.

In parallel, research on exceptional longevity populations, including so-called “Blue Zones”, has highlighted the potential role of modifiable lifestyle, environmental, and social factors in promoting healthy aging. The Cilento region in southern Italy represents one such context, characterized by a high prevalence of long-lived individuals and distinctive lifestyle patterns. Previous studies conducted by the present author (Aliberti et al., 2022; Aliberti et al., 2022b; Aliberti et al., 2024) [17,18,19] have described the demographic, environmental, nutritional, and social characteristics of this population, suggesting that adherence to traditional dietary patterns, sustained physical activity, and strong social cohesion may contribute to favorable aging trajectories. However, research on the Cilento and other Blue Zones has so far relied on epidemiological and clinical data, without systematic quantification of the underlying epigenetic mechanisms.

To date, no systematic review and meta-analysis has comprehensively synthesized and standardized the association between modifiable determinants and epigenetic age acceleration using validated DNA methylation clocks (Horvath, PhenoAge, GrimAge, DunedinPACE), nor quantified the magnitude of these effects across domains within a unified framework. This gap represents a critical barrier to integrating insights from geroscience into preventive medicine and public health. In addition to the lack of a unified synthesis, no previous study has attempted to quantify the cumulative and domain-specific burden of modifiable determinants on biological aging. Existing literature has generally focused on isolated exposures, without providing an integrated metric capable of summarizing their combined impact on epigenetic age acceleration. This limitation hampers the translation of molecular epidemiology findings into actionable public health and preventive medicine frameworks. To address this gap, we developed the Modifiable Epigenetic Aging Burden Index (MEAB-Index), a composite measure designed to aggregate pooled effect estimates across exposure categories and to estimate the potentially preventable component of epigenetic aging attributable to modifiable factors. Conceptually, the MEAB-Index aims to bridge molecular epidemiology with population health and prevention-oriented geroscience by quantifying the collective contribution of behavioral, environmental, and social determinants to biological aging trajectories.

The present study aims to address this gap by conducting a systematic review and meta-analysis of observational and interventional studies examining the associations between modifiable behavioral, environmental, and social determinants and EAA in adult populations. Specifically, we aim to: (i) systematically identify studies assessing EAA in relation to modifiable determinants; (ii) harmonize effect estimates into a common metric to enable comparability across studies; (iii) quantify the average magnitude and direction of these associations, including subgroup analyses by determinant category and epigenetic clock type. In doing so, this work builds upon previous research on the Cilento and other Blue Zones populations, transitioning from epidemiological observation toward a quantitative assessment of the biological pathways through which modifiable factors may shape epigenetic aging.

By conceptualizing EAA as a modifiable and potentially preventable outcome, this study seeks to bridge the gap between molecular biomarkers of aging and population-level prevention strategies, providing a quantitative framework to inform interventions aimed at promoting healthy aging and reducing the burden of age-related diseases.

## 2. Materials and Methods

### 2.1. Protocol and Reporting Standards

This systematic review and meta-analysis was conducted and reported in accordance with the Preferred Reporting Items for Systematic Reviews and Meta-Analyses (PRISMA) 2020 statement [20]. The study protocol was registered in PROSPERO (CRD420261371271) [21] following completion of the literature search and study selection, and prior to data extraction and quantitative synthesis.

Minor deviations from the registered protocol were introduced to refine the analytical framework. These included a modest refinement of the study title to better reflect the final scope and emphasis of the work, as well as the development and inclusion of the Modifiable Epigenetic Aging Burden Index (MEAB-Index) to quantify the cumulative preventable burden of epigenetic age acceleration. All deviations were defined and documented prior to the start of quantitative data synthesis and did not affect the pre-specified eligibility criteria or primary outcomes.

### 2.2. Research Question and Objective

The primary research question was: What is the magnitude and direction of the association between modifiable behavioral, environmental, and social determinants and epigenetic age acceleration (EAA) measured using validated DNA methylation-based clocks in adult populations?

The study had three operational objectives: (i) to systematically identify and synthesize all relevant studies examining associations between modifiable determinants and epigenetic aging; (ii) to standardize heterogeneous effect estimates into a common, interpretable metric, primarily expressed as years of EAA; and (iii) to quantify the overall impact of modifiable determinants on biological aging and derive a composite measure of preventable public health burden, defined as the Modifiable Epigenetic Aging Burden Index (MEAB-Index).

### 2.3. Eligibility Criteria (PICOS Framework)

Eligibility criteria were defined a priori according to the PICOS framework:

○ Population: Adults (≥18 years) from the general population or community-based samples.
○ Exposure: Modifiable behavioral, environmental, psychosocial, or socioeconomic determinants (e.g., lifestyle factors, diet, physical activity, smoking, alcohol consumption, sleep, stress, environmental exposures, socioeconomic conditions).
○ Comparator: Different levels of exposure or reference/unexposed groups.
○ Outcome: Epigenetic age acceleration (EAA), defined as the residual from regressing epigenetic age on chronological age, measured using validated DNA methylation-based clocks (i.e., Horvath, Hannum, PhenoAge, GrimAge/GrimAge2, DunedinPoAm, DunedinPACE).
○ Study design: Observational studies (cross-sectional, cohort, case–control) and interventional studies reporting quantitative associations.

Exclusion criteria included studies conducted in pediatric populations, animal models, reviews, editorials, protocols, methodological-only studies (e.g., clock development without exposure–outcome association), and studies lacking extractable quantitative data.

### 2.4. Information Sources and Search Strategy

A comprehensive literature search was conducted in PubMed/MEDLINE and Scopus from database inception to 7 April 2026. Europe PMC was additionally searched to enhance retrieval of open-access publications.

The search strategy was structured around three conceptual domains: (i) epigenetic aging measures, (ii) modifiable determinants, and (iii) study design and association terms. Within each domain, terms were combined using the Boolean operator OR, and the three domains were combined using AND.

The epigenetic aging domain included terms such as “epigenetic clock”, “DNA methylation age”, “epigenetic age acceleration”, “biological age”, and specific clock names (Horvath, Hannum, PhenoAge, GrimAge, DunedinPoAm, DunedinPACE). The modifiable determinants domain included lifestyle, behavioral, environmental, and socioeconomic exposures (e.g., diet, physical activity, smoking, alcohol consumption, sleep, psychosocial stress, air pollution, and socioeconomic status). The study design domain included terms such as association, regression, observational, cohort, cross-sectional, case–control, and randomized controlled trial.

The full electronic search strategy for PubMed is reported below and was adapted for the other databases:

(“epigenetic clock” [Title/Abstract] OR “DNA methylation age” [Title/Abstract] OR “epigenetic age” [Title/Abstract] OR “biological age” [Title/Abstract] OR “epigenetic age acceleration” [Title/Abstract] OR “age acceleration” [Title/Abstract] OR Horvath [Title/Abstract] OR Hannum [Title/Abstract] OR PhenoAge [Title/Abstract] OR GrimAge [Title/Abstract] OR DunedinPoAm [Title/Abstract] OR DunedinPACE [Title/Abstract]) AND (diet [Title/Abstract] OR nutrition [Title/Abstract] OR “physical activity” [Title/Abstract] OR exercise [Title/Abstract] OR smoking [Title/Abstract] OR alcohol [Title/Abstract] OR sleep [Title/Abstract] OR stress [Title/Abstract] OR “air pollution” [Title/Abstract] OR “environmental exposure*” [Title/Abstract] OR “socioeconomic status” [Title/Abstract]) AND (association [Title/Abstract] OR regression [Title/Abstract] OR observational [Title/Abstract] OR cohort [Title/Abstract] OR “cross-sectional” [Title/Abstract] OR “case–control” [Title/Abstract] OR “randomized controlled trial” [Title/Abstract]).

No language or geographical restrictions were applied, except that only articles published in English were considered eligible. Additional studies were identified through manual screening of reference lists of included studies and relevant reviews, as well as forward citation tracking via Google Scholar. No search updates were performed after 7 April 2026.

### 2.5. Study Selection

Study selection was performed in two stages: (i) screening of titles and abstracts, and (ii) full-text assessment. To enhance efficiency and reproducibility while minimizing bias, a semi-automated Python-based workflow (Python 3.14.5, Python Software Foundation, Wilmington, DE, USA, 2023) was used exclusively for deduplication, data organization, and preliminary keyword-based flagging of records. This tool served only as decision support; no records were automatically excluded and it had no influence on final inclusion or exclusion decisions.

All records were independently screened by two reviewers (S.M.A. and P.M.). Each record was classified as “include”, “exclude”, or “unclear” based on the pre-defined eligibility criteria. Disagreements were resolved by discussion and consensus, with consultation of a third reviewer when necessary. Reasons for exclusion at the title and abstract stage were recorded using pre-specified categories: (i) wrong outcome (not epigenetic age or epigenetic age acceleration), (ii) wrong exposure (not a modifiable determinant), (iii) wrong population (e.g., pediatric-only populations, animal studies), (iv) wrong study design (e.g., review, editorial, protocol), (v) methodological-only studies (e.g., clock development without exposure–outcome analysis), and (vi) other reasons.

**Figure 1:**
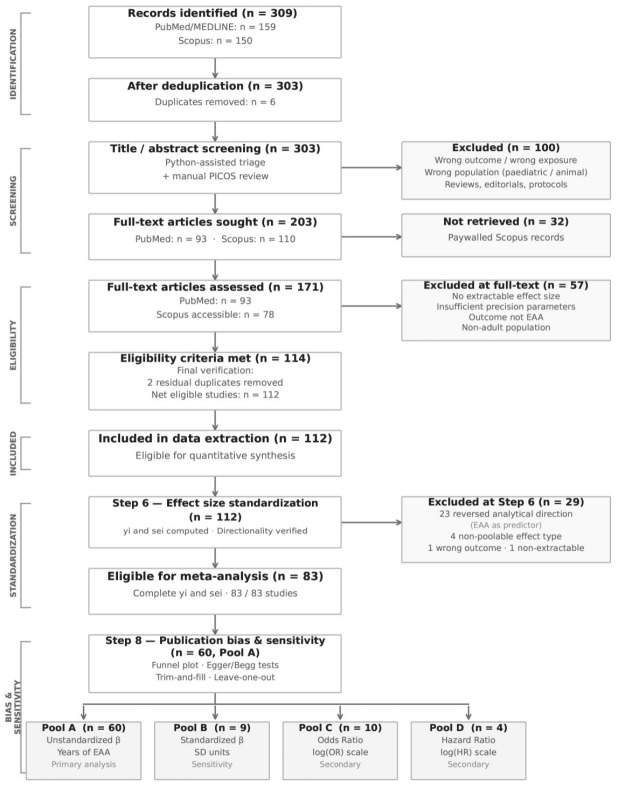
PRISMA 2020 flow diagram of the study selection process for the systematic review and meta-analysis.

Potentially eligible studies underwent full-text assessment by the same two reviewers working independently. Reasons for exclusion at the full-text stage were documented in detail. The study selection process is presented in a PRISMA flow diagram (Figure 1). Of the records selected for full-text assessment, most articles were successfully retrieved through institutional subscriptions and open-access sources; however, a subset of records could not be accessed in full-text form despite repeated retrieval attempts via institutional library services, publisher platforms, and open-access repositories, and therefore did not provide extractable quantitative data.

### 2.6. Data Extraction

Data extraction was performed using a pre-piloted, standardized data collection form developed specifically for this review. The following information was extracted from each included study: study identifiers (first author, year, journal), study design, population characteristics (sample size, mean age, sex distribution), detailed definition and measurement of the exposure(s), type of DNA methylation-based epigenetic clock used, definition and operationalization of epigenetic age acceleration (EAA), effect estimates (including beta coefficients, mean differences, standardized mean differences, odds ratios, hazard ratios), corresponding measures of uncertainty (95% confidence intervals, standard errors, and p-values), statistical modelling approaches, and covariates adjusted for in multivariable models. Geographical classification of studies was based on the location of the study population (i.e., where data were collected), rather than on author affiliations.

To facilitate data organization and initial harmonization, a semi-automated Python-based pipeline was used. However, all extracted data were independently verified and manually checked for accuracy by the reviewers. Data extraction was conducted independently by two reviewers (S.M.A. and P.M.). Any discrepancies were resolved through discussion and, when necessary, consultation with a third reviewer. To further ensure data quality and internal consistency, an additional duplicate data verification was performed on a random subset of 20% of the included studies, with consistency checks applied across key variables.

### 2.7. Eligibility for Quantitative Synthesis and Effect Size Harmonization

Studies were eligible for quantitative synthesis if they reported a quantitative association between modifiable determinants and epigenetic age acceleration (EAA) and provided sufficient information to calculate effect sizes and their measures of variance.

Due to expected heterogeneity in effect metrics across studies, a pre-specified harmonization framework was applied. Effect estimates were classified into four mutually exclusive analytical pools based on their statistical format:

○ Pool A (primary analysis): Unstandardized beta coefficients or mean differences expressed in years of epigenetic age acceleration (EAA).
○ Pool B (sensitivity analysis): Standardized beta coefficients or standardized mean differences (SMD).
○ Pool C: Odds ratios (OR).
○ Pool D: Hazard ratios (HR).

For Pool A, the effect size (y_i_) was taken directly as the reported beta coefficient or mean difference. When standard errors were not reported, they were derived from 95% confidence intervals using the formula:

$$SE=\frac{CI_{upper}-CI_{lower}}{3.92}$$

For Pools C and D, effect estimates were log-transformed (y_i_ = In(OR) or In(HR)), and corresponding standard errors were calculated from the reported confidence intervals.

When exposures were reported on different scales (e.g., per 1-unit increase, per standard deviation, per interquartile range, or as categorical contrasts), effect sizes were harmonized to a common scale using pre-specified conversion rules to maximize comparability. Studies that did not provide adequate data for harmonization, as well as exposure categories not meeting the minimum threshold for quantitative synthesis (k ≥ 3), were included in the qualitative synthesis but excluded from quantitative meta-analysis.

#### Rationale for Combining Different Epigenetic Clocks

Although the included studies used different DNA methylation-based clocks, these measures capture overlapping, although not identical, dimensions of biological aging and are therefore considered complementary indicators of cumulative biological burden. For this reason, epigenetic age acceleration (EAA) was treated as a unified outcome in the primary pooled analyses. To account for potential differences in sensitivity and predictive domains across clocks, clock type was included as a moderator in pre-specified subgroup and meta-regression analyses. This approach allowed us to evaluate the contribution of clock type to between-study heterogeneity and to interpret both pooled and clock-specific findings with appropriate caution.

### 2.8. Statistical Analysis

Meta-analyses were performed using a random-effects model, which assumes that true effects may vary across studies and therefore accounts for expected between-study heterogeneity in populations, exposure definitions, and analytical approaches. Between-study variance (τ^2^), representing the amount of true heterogeneity, was estimated using the DerSimonian–Laird method [22].

Pooled effect estimates (θ) were calculated as inverse-variance weighted averages, giving greater weight to studies with more precise estimates (i.e., smaller standard errors):

$$w_{i}=\frac{1}{v_{i}+\tau^{2}}$$

where $w_{i}$ is the weight assigned to study i, and $v_{i}\;$ is the within-study variance.

Statistical heterogeneity was assessed using Cochran’s Q test (*p* < 0.10 considered statistically significant) and quantified with the I^2^ statistic, which expresses the proportion of variability due to true heterogeneity rather than chance, and with τ^2^.

Pre-specified subgroup analyses were conducted according to exposure categories of modifiable determinants (e.g., smoking, diet, physical activity, socioeconomic status), where applicable.

Meta-regression analyses, which evaluate whether study-level characteristics explain variability in effect sizes, were performed to explore potential sources of heterogeneity, including study design, population characteristics, type of epigenetic clock, and level of covariate adjustment.

The robustness of the pooled estimates was evaluated through sensitivity analyses, including: (1) restriction to studies with full multivariable adjustment, (2) exclusion of studies with high risk of bias, and (3) removal of influential studies identified through leave-one-out analysis and Cook’s distance. Publication bias was assessed visually using funnel plots and statistically using Egger’s test (when ≥10 studies were available).

### 2.9. Development of the MEAB-Index

To quantify the cumulative impact of modifiable determinants on biological aging, we developed the Modifiable Epigenetic Aging Burden Index (MEAB-Index) based on pooled estimates derived from the primary meta-analysis (Pool A).

Three complementary metrics were defined:

1. MEAB-composite: The overall burden across all exposure categories, calculated as an inverse-variance weighted average of the category-level pooled effect estimates:
  $$\mathrm{MEAB-composite}=\frac{\sum (w_{k}\cdot \beta_{k})}{\sum w_{k}}$$
  where $w_{k}=\frac{1}{SE_{k}^{2}}$, and $\beta_{k}$ and $SE_{k}$ represent the pooled effect size and its standard error for exposure category *k*, respectively. Category-level estimates were combined using a fixed-effect framework. The standard error of the MEAB-composite was computed as $\mathrm{SE}(\mathrm{MEAB-composite})=1/\sqrt{\sum w_{k}}$
2. MEAB-positive: Calculated using the same inverse-variance weighted approach but restricted to exposure categories that showed statistically significant positive association with epigenetic age acceleration (EAA, *p* < 0.05).
3. Cumulative Preventable Burden (CPB): The unweighted sum of all statistically significant positive pooled estimates:
  $$\mathrm{CPB}=\sum_{k\in S}\beta_{k}$$
  where S is the set of exposure categories with significant positive associations. The CPB represents the theoretical maximum reduction in EAA achievable by eliminating all identified risk factors and should be interpreted as an upper-bound estimate, given potential correlations and overlaps between exposures.

Uncertainty for all three metrics was quantified using non-parametric bootstrap resampling (10,000 iterations). Category-level pooled estimates were sampled from normal distributions $N\left(\beta_{k},{\mathrm{SE}}_{k}^{2}\right)$ and percentile-based 95% confidence intervals were derived. Sensitivity analyses were performed using a leave-one-category-out approach to evaluate the influence of individual exposures on index stability.

### 2.10. Risk of Bias and Certainty of Evidence

Risk of bias was assessed separately according to study design. For observational studies, the Newcastle-Ottawa Scale (NOS) was used. For randomized or interventional studies (if any), the Cochrane Risk of Bias 2 (RoB 2) tool was applied.

Two reviewers (S.M.A. and P.M.) independently evaluated the risk of bias, with disagreements resolved by discussion and consensus.

Publication bias was examined through visual inspection of funnel plots and formally tested using Egger’s regression test and Begg’s rank correlation test. When at least 10 studies were available for a given exposure, the trim-and-fill method was used to explore the potential impact of small-study effects.

The overall certainty of the body of evidence for each exposure category was rated using the Grading of Recommendations Assessment, Development and Evaluation (GRADE) framework. The five domains evaluated were: risk of bias, inconsistency, indirectness, imprecision, and publication bias. The certainty of evidence was graded as high, moderate, low, or very low.

### 2.11. Reproducibility and Software

All statistical analyses were conducted using R version 4.3.2 (R Core Team, R Foundation for Statistical Computing, Vienna, Austria, 2024) [23] primarily through the metafor package [24] and Python 3.14.5 (Python Software Foundation, Wilmington, DE, USA, 2023) [25].

The complete analytical workflow, including data cleaning, harmonization, meta-analysis, MEAB-Index calculation, and sensitivity analyses, was predefined within a scripted pipeline to ensure computational reproducibility.

To further enhance transparency and reproducibility, all R and Python scripts used for data cleaning, harmonization, meta-analytic modelling, MEAB-Index computation, and sensitivity analyses are openly available in the dedicated GitHub repository: https://github.com/smaliberti/eaa-meta-analysis (accessed on 28 May 2026).

All R and Python scripts are available from the corresponding author upon reasonable request.

## 3. Results

### 3.1. Study Selection

The study selection process is presented in Figure 1, following the PRISMA 2020 guidelines.

A total of 309 records were identified through database searching (PubMed/MEDLINE: *n* = 159; Scopus: *n* = 150). After removal of 6 duplicates, 303 unique records were screened at the title and abstract level. Of these, 203 records were considered potentially eligible and advanced to full-text assessment. Full texts were successfully retrieved for 171 articles, while the remaining records lacked an accessible full-text version despite retrieval attempts. Following full-text evaluation, 57 studies were excluded due to predefined criteria. Consequently, 114 studies initially met the inclusion criteria. After removal of 2 residual duplicates identified during data extraction, 112 unique studies were retained.

All studies underwent effect size harmonization. During this phase, 29 studies were excluded from quantitative synthesis due to incompatible analytical direction (EAA used as predictor rather than outcome), non-poolable effect measures, or insufficient data for variance estimation. Ultimately, 83 studies providing 118 distinct exposure–clock associations were included in the meta-analysis.

These studies were classified into four analytical pools according to the reported effect metrics: Pool A (unstandardized beta coefficients or mean differences, *n* = 60 studies), Pool B (standardized beta coefficients or SMD, *n* = 9), Pool C (odds ratios, *n* = 10), and Pool D (hazard ratios, *n* = 4). Detailed results for each analytical pool are presented in Supplementary Tables S1–S4. The main characteristics of all included studies are summarized in Table 1, while a summary of the pooled effect sizes across the four pools is presented in Table 2.

### 3.2. Overall Characteristics of Included Studies

More than half of the studies were conducted in North America (57.8%), predominantly in the United States, followed by Europe (25.3%) and Asia/Other regions (16.9%). Cross-sectional designs were the most common (50.6%), followed by prospective cohort studies (34.9%). The median sample size was 8742 participants (range 158–343,723), with a mean participant age of 56.4 years. Fully adjusted models were used in 79.5% of the studies.

Lifestyle and behavioral factors represented the largest exposure category (42.2%), followed by environmental exposures (22.9%) and socioeconomic/psychosocial determinants (19.3%). Second- and third-generation clocks were predominant, with GrimAge/GrimAge2 (31.3%) and PhenoAge (25.3%) being the most frequently used.

#### 3.2.1. Primary Analysis: Pool A (Unstandardized Effect Sizes, *n* = 60 Studies)

In the primary analysis (Pool A), the overall random-effects model yielded a pooled β = +0.310 years of EAA per unit increase in adverse exposure (95% CI: +0.255 to +0.366; z = 10.96, *p* < 0.001). Adverse modifiable exposures were consistently associated with accelerated biological aging, while protective factors showed deceleration.

**Figure 2:**
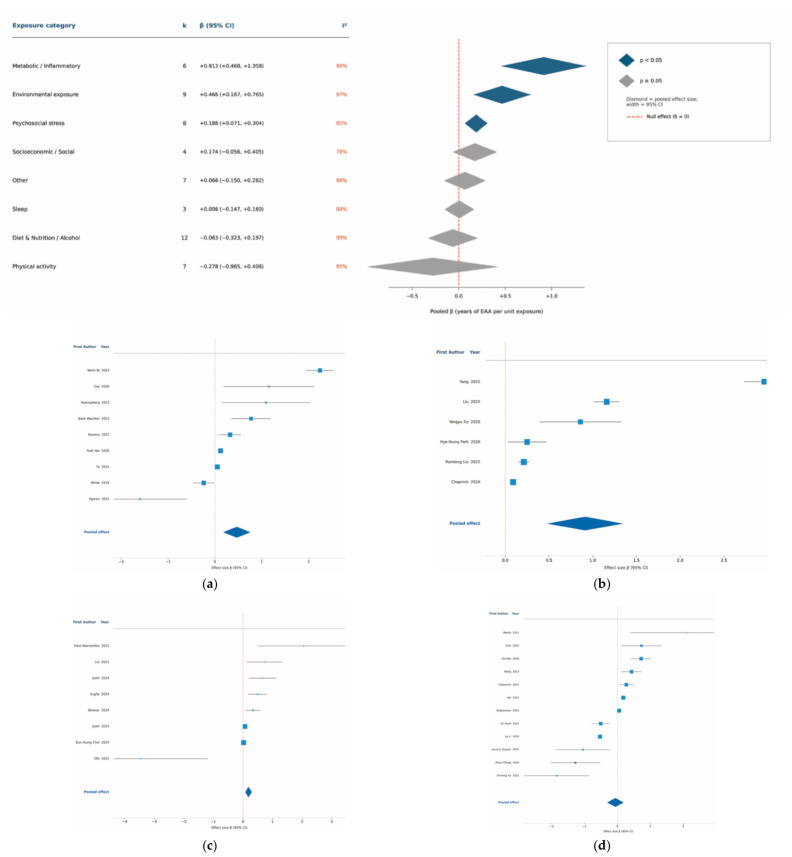

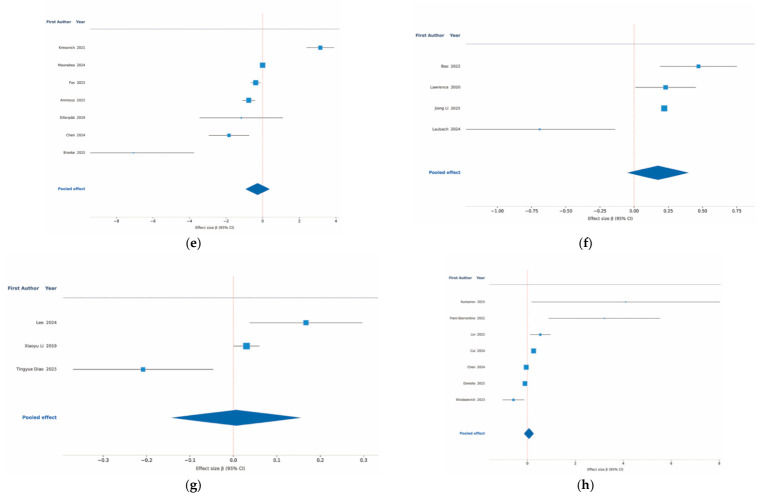
Summary forest plot of pooled estimates by exposure category (Pool A, random-effects model). Each diamond represents the pooled β for the category, with horizontal lines indicating the 95% confidence interval. The overall pooled estimate is shown at the bottom. Individual forest plots for each exposure category (**a**–**h**) use category-specific axis scales because study-level effect sizes vary substantially across exposure domains; therefore, cross-category comparisons should rely on the pooled summary forest plot. The red dashed vertical line represents the null value (β = 0.0). (**a**) Environmental exposures: Wenli Ni, 2023 [37]; Zuo, 2026 [38]; Koenigsberg, 2023 [39]; Blechter, 2023 [40]; Baranyi, 2022 [41]; Yueli Yao, 2026 [42]; Yu, 2025 [43]; White, 2019 [44]; Egorov, 2025 [45]. (**b**) metabolic and inflammatory markers: Yang, 2025 [46]; Liu, 2025 [47]; Yangyu Xu, 2026 [48]; Hye-Young Park, 2026 [49]; Rundong Liu, 2025 [50]; Chapnick, 2026 [51]. (**c**) Psychosocial stress: Freni-Sterrantino, 2022 [52]; Liu, 2022 [53]; Joshi, 2024 [54]; Suglia, 2024 [55]; Skinner, 2024 [56]; Joshi, 2023 [57]; Eun Young Choi, 2024 [58]; Ohi, 2025 [59]. (**d**) dietary/nutritional exposures: Martin, 2021 [60]; Choi, 2025 [61]; Kai Wei, 2026 [62]; Wang, 2023 [63]; Kresovich, 2021 [64]; Xia, 2025 [65]; Stephenson, 2021 [66]; Fei Shan, 2025 [67]; Jie Li, 2025 [68]; Bozack, 2025 [69]; Xinyu Zhang, 2026 [70]; Xinming Xu, 2025 [71]. (**e**) Physical activity: Kresovich, 2021 [72]; Maunakea, 2024 [73]; Fox, 2023 [74]; Ammous, 2025 [75]; Sillanpää, 2019 [76]; Chen, 2024 [77]; Brooke, 2025 [78]. (**f**) socioeconomic/social factors: Bao, 2022 [79]; Lawrence, 2020 [80]; Jiang Li, 2025 [81]; Laubach, 2024 [82]. (**g**) Sleep-related exposures: Lee, 2024 [83]; Xiaoyu Li, 2019 [84]; Tingyue Diao, 2025 [85]. (**h**) Other exposures: Kurbanov, 2025 [33]; Freni-Sterrantino, 2022 [36]; Lin, 2025 [34]; Cui, 2024 [30]; Chen, 2024 [31]; Daredia, 2025 [35]; Khodasevich, 2023 [32].

Substantial between-study heterogeneity was observed (I^2^ = 98.8%, τ^2^ = 0.024). Subgroup analyses by exposure category are presented in Table 3 and Figure 2. The forest plot illustrates a high degree of consistency in the direction of effects across studies, despite variability in effect magnitude.

Of the 60 included studies, 49 met criteria for quantitative pooling across seven predefined exposure domains. The remaining 11 studies were excluded from meta-analysis due to insufficient category size (k < 3) or lack of conceptual comparability but were retained for narrative synthesis. These studies encompassed heterogeneous exposures, including mixed lifestyle profiles [26,27], smoking-related exposures across the life course [28,29,30], cognitive function [31], prenatal environmental toxicants [32], disease-specific conditions [33,34], reproductive aging [35], and isolated environmental toxicants [36].

Environmental exposures (k = 9) were significantly associated with epigenetic age acceleration (β of +0.466 years, 95% CI: +0.167 to +0.765, *p* = 0.002), consistent with studies on air pollution, chemical mixtures and temperature [37,38,39,40,41,42,43,44,45].

Metabolic and inflammatory markers showed the largest effect size (k = 6, β = +0.913 years, 95% CI: +0.468 to +1.358, *p* < 0.001), highlighting the central role of systemic inflammation and cardiometabolic dysregulation in epigenetic aging, in line with the inflammaging paradigm [5,86] and recent empirical findings [46,47,48,49,50,51].

Psychosocial stress (k = 8) was also significantly associated with greater epigenetic age acceleration (β = +0.188 years, 95% CI: +0.071 to +0.304, *p* = 0.002), with associations persisting even after adjustment for behavioral mediators [52,53,54,55,56,57,58,59].

In contrast, diet and nutrition (k = 12) [60,61,62,63,64,65,66,67,68,69,70,71] showed a protective trend (β = −0.063 years), but no statistically significant association was observed. Physical activity (k = 7) [72,73,74,75,76,77,78] also showed protective trends (−0.278 years), but no statistically significant association was observed. Socioeconomic and social factors (k = 4) [79,80,81,82] showed a positive trend (*p* = 0.139), but no statistically significant association was observed. Sleep (k = 3) [83,84,85] showed no statistically significant association (β = +0.006 years, 95% CI: −0.147 to +0.160, *p* = 0.936).

Categories with k < 3 were retained only for narrative synthesis.

To enhance transparency and allow evaluation of study-level contributions, individual forest plots for each exposure category have been added as Figure 2a–h, displaying the effect sizes and corresponding weights of all included studies. Because the magnitude of study-level effects varies substantially across exposure categories, the individual forest plots (Figure 2a–h) use category-specific axis scales. Cross-category comparisons should therefore rely on the pooled summary forest plot rather than on the individual panels.

Meta-regression analyses identified exposure category and epigenetic clock type as significant moderators of the association, partially explaining between-study variability, whereas study design and level of covariate adjustment showed more limited influence.

#### 3.2.2. Publication Bias and Sensitivity Analyses (Pool A)

**Figure 3:**
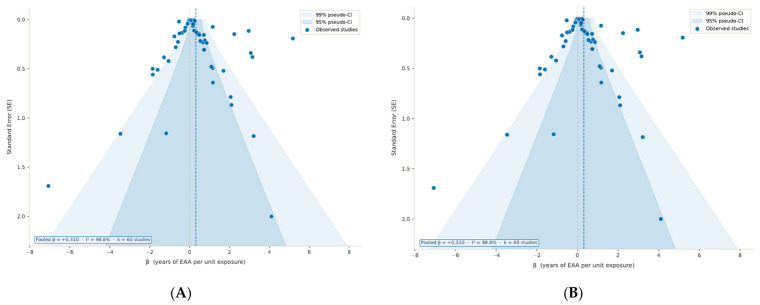
Assessment of publication bias in the primary analysis (Pool A, *n* = 60 studies). The red dashed line indicates the null value (β = 0), while the vertical dashed blue line represents the pooled estimate (β = +0.310). (**A**) Standard funnel plot. The *x*-axis shows the effect size β (years of epigenetic age acceleration per unit increase in adverse exposure)). The *y*-axis shows the standard error (inverted). Blue dots represent the observed studies. Shaded areas represent the 95% (darker) and 99% (lighter) pseudo-confidence intervals. (**B**) Funnel plot with trim-and-fill correction (Duval & Tweedie method). No studies were imputed (k = 0).

**Figure 4:**
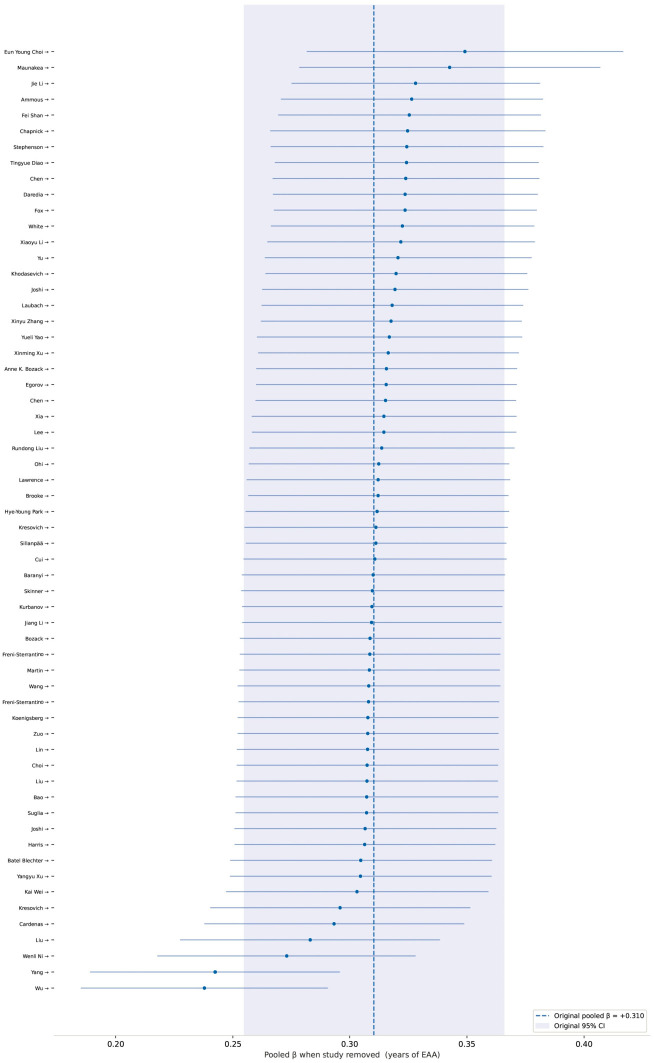
Leave-one-out sensitivity analysis for Pool A (*n* = 60 studies) [26,27,28,29,30,31,32,33,34,35,36,37,38,39,40,41,42,43,44,45,46,47,48,49,50,51,52,53,54,55,56,57,58,59,60,61,62,63,64,65,66,67,68,69,70,71,72,73,74,75,76,77,78,79,80,81,82,83,84,85,86]. Each horizontal line represents the pooled β estimate and 95% confidence interval obtained after the exclusion of one study. Studies are ordered from the highest (top) to the lowest (bottom) resulting pooled estimate (top to bottom). The vertical dashed blue line indicates the original pooled estimate (β = +0.310 years of EAA), with its 95% confidence interval shown as the shaded band. Blue dots indicate statistically significant pooled estimates (*p* < 0.05).

Publication bias was visually and statistically assessed in Pool A. Figure 3 presents the funnel plots for the primary analysis. Figure 3A shows the standard funnel plot, while Figure 3B displays the trim-and-fill corrected plot. Egger’s test (*p* < 0.001) and Begg’s test (*p* = 0.058) suggested potential asymmetry. However, the trim-and-fill method (Figure 3B) identified no missing studies (k = 0), and the adjusted pooled estimate remained virtually unchanged (β = +0.310 years). Leave-one-out sensitivity analysis (Figure 4) confirmed high robustness: the pooled β ranged from +0.238 to +0.349 and remained statistically significant in all iterations.

#### 3.2.3. The MEAB-Index: Quantifying the Preventable Burden of Epigenetic Aging

**Figure 5:**
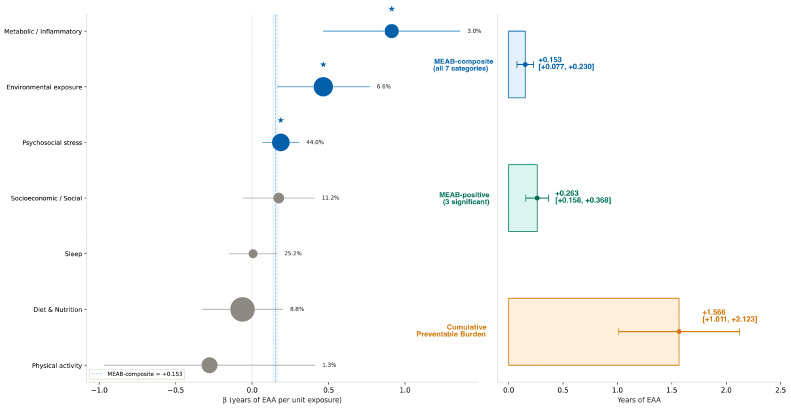
MEAB-Index component chart. Left panel: Pooled β coefficient (years of EAA per unit exposure) for each exposure category. Dot size is proportional to the number of studies (k); ★ indicates statistically significant associations (*p* < 0.05). The dashed blue vertical line represents the MEAB-composite value. Right panel: Summary of three MEAB-Index metrics with bootstrap 95% confidence interval. Blue = MEAB-composite (all seven categories); teal = MEAB-positive (only significant categories); amber = Cumulative Preventable Burden (CPB).

**Figure 6:**
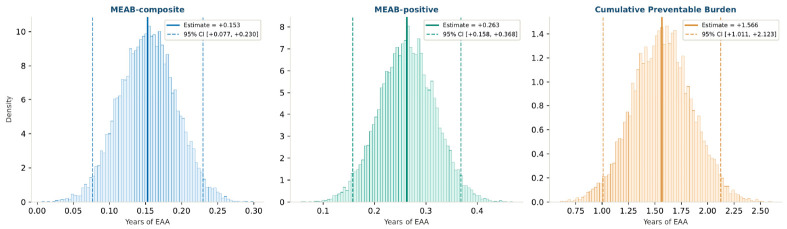
Bootstrap distributions of the three MEAB-Index metrics (10,000 iterations). The vertical solid line represents the point estimate, and dashed lines indicate the 95% bootstrap confidence intervals. The shaded area corresponds to the 95% confidence region. From left to right: MEAB-composite, MEAB-positive, and Cumulative Preventable Burden (CPB).

**Figure 7:**
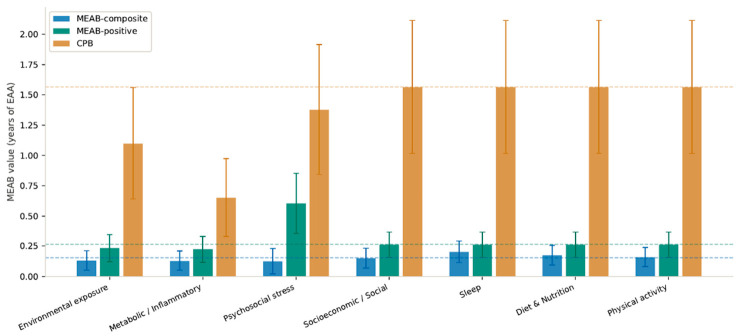
Sensitivity analysis of the MEAB-Index by sequential removal of one exposure category at a time. Blue bars = MEAB-composite; teal bars = MEAB-positive; amber bars = Cumulative Preventable Burden (CPB). Error bars represent 95% bootstrap confidence intervals. Dashed horizontal lines indicate the values obtained with the full model (all categories included).

The Modifiable Epigenetic Aging Burden Index (MEAB-Index) was computed to synthesize category-specific effects into public health-relevant metrics (Table 4, Figure 5, Figure 6 and Figure 7).

The Cumulative Preventable Burden (CPB) was estimated at +1.566 years (bootstrap 95% CI: +1.011 to +2.123), representing the theoretical maximum reduction in epigenetic aging achievable by eliminating the three statistically supported risk factors (environmental exposures, metabolic/inflammatory dysregulation, and psychosocial stress).

#### 3.2.4. Secondary Analyses (Pools B–D)

Results from secondary analytical pools (B, C and D) [87,88,89,90,91,92,93,94,95,96,97,98,99,100,101,102,103,104,105,106,107,108] were directionally consistent with the primary analysis, reinforcing the robustness of the observed associations across different effect size metrics and analytical frameworks (Supplementary Tables S2–S4).

## 4. Discussion

### 4.1. Overview and Interpretation of Findings

This systematic review and meta-analysis provides a comprehensive and quantitatively integrated synthesis of the associations between modifiable environmental, behavioral, and social determinants and epigenetic age acceleration (EAA), positioning biological aging as a measurable and potentially modifiable outcome of the exposome. By integrating 83 studies and 118 distinct exposure–clock associations across four complementary analytical pools (A–D), the present work provides robust evidence that adverse exposures are consistently associated with accelerated biological aging, whereas protective factors show directional trends toward deceleration. Collectively, these findings support the conceptualization of epigenetic aging as a dynamic, biologically embedded, yet modifiable process.

Environmental exposures emerged as a major domain influencing EAA. Multiple lines of evidence converged on the detrimental impact of air pollution and chemical mixtures. Studies focusing on ambient fine particulate matter and nitrogen dioxide [39,41,42,44] consistently documented accelerated EAA, likely mediated by oxidative stress, systemic inflammation, and direct epigenetic modifications at loci regulating inflammation and DNA repair. These associations were reinforced by research on household air pollution from solid fuel combustion [40] and complex occupational or environmental chemical mixtures [38], suggesting that the cumulative burden of diverse pollutants may exert additive or synergistic effects on the epigenome. Temperature-related exposures further highlighted the sensitivity of biological aging to climatic factors. Both short- and long-term temperature variability [37,43] were associated with increased EAA, possibly through mechanisms involving heat shock proteins, inflammatory responses, and disruption of circadian rhythms. In contrast, protective environmental features showed clear beneficial effects. Residential greenness [45] was linked to slower epigenetic aging, an effect plausibly explained by reduced exposure to pollutants, lower psychological stress, increased physical activity, and enhanced microbial diversity, all factors known to modulate inflammatory pathways.

A particularly noteworthy aspect concerns the potential protective role of mild and stable climate conditions. Regions characterized by temperate, Mediterranean-type climates with limited extreme temperature fluctuations—such as the Cilento area in southern Italy, a candidate Blue Zone—may contribute to slower epigenetic aging trajectories. This hypothesis aligns with the present findings on temperature variability as a risk factor and is supported by previous work from the present research group (Aliberti et al., 2022a, 2023, 2025a) [17,109,110], where mild climate, mineral-rich water, and low exposure to thermal extremes coexist with exceptional healthspan. The convergence between these population-level observations and the molecular evidence from the current meta-analysis suggests that stable, non-extreme climatic conditions may represent an underappreciated protective environmental factor, possibly by minimizing chronic activation of stress-response systems and preserving epigenetic stability over the life course.

Metabolic and inflammatory markers exhibited some of the strongest and most consistent associations with accelerated EAA. This domain included complementary indicators of cardiometabolic dysfunction and systemic inflammation. Robust associations were observed for the cardiometabolic index [47], metabolic syndrome severity [49], and metabolic dysfunction-associated steatotic liver disease [46]. These metabolic findings were reinforced by studies focusing on direct inflammatory burden, particularly the systemic immune-inflammation index [50] and neutrophil-related inflammatory markers [48]. Collectively, this body of evidence strongly supports the inflammaging paradigm [5,86] and the exposome-driven pathobiological framework previously outlined by the present author (Aliberti 2025b) [111]. Within this model, metabolic and inflammatory disturbances represent critical nodes where environmental, behavioral, and social exposures converge to accelerate biological aging. The findings mirror the lower burden of cardiometabolic and inflammatory dysregulation documented in Cilento and Sicilian Mountain villages, where traditional Mediterranean dietary patterns and active lifestyles appear to mitigate these pathways.

Psychosocial stress demonstrated robust and largely independent associations with greater epigenetic age acceleration. This domain encompassed a broad spectrum of stressors operating at different life stages and ecological levels. Adverse childhood experiences [57] were strongly linked to faster EAA. These long-term effects were complemented by evidence on adult psychosocial burden, including general psychosocial stress [55,56], clinical depression [53], anxiety disorders [59], work-related stress and job strain [52], and neighborhood-level social stressors and deprivation [54,58]. A key strength of this body of evidence is the persistence of associations even after adjustment for behavioral mediators. This suggests that psychosocial stress influences the epigenome through direct biological pathways, including sustained activation of the hypothalamic–pituitary–adrenal (HPA) axis, sympathetic nervous system overdrive, and pro-inflammatory signaling. The consistency across different constructs—from early-life trauma to chronic adult stress and neighborhood disadvantage—supports a life-course model of psychosocial embedding. These mechanisms align with the Brain–Energy–Microbiome–Exposome framework previously proposed by the present author (Aliberti et al., 2025c) [112], whereby chronic psychosocial adversity contributes to accelerated biological aging through interconnected neuroendocrine, inflammatory, and epigenetic pathways, ultimately influencing healthspan trajectories observed in long-lived populations such as those in the Cilento region.

Dietary and nutritional factors and physical activity generally showed protective associations. Higher diet quality, Mediterranean-type patterns, and beneficial nutrients such as polyunsaturated fatty acids [67] and vitamin intake [70] tended to be associated with slower EAA. Similarly, leisure-time physical activity [76], objectively measured activity [74], and life-course physical activity trajectories [75,77] were linked to reduced epigenetic aging. These protective effects align closely with the lifestyle characteristics observed in Cilento and other Blue Zones, where traditional Mediterranean diets and sustained physical activity linked to daily life are considered central pillars of exceptional longevity (Aliberti et al., 2022, 2024) [17,19].

Although these domains consistently showed protective trends, no statistically significant associations were observed in the pooled estimates. This lack of statistical significance likely reflects the heterogeneity of exposure assessment methods, variability in measurement instruments (e.g., self-reported vs. objective physical activity), and the limited number of available studies in some categories. Importantly, the directionality of effects remained coherent across studies, suggesting that the absence of statistical significance may be attributable to methodological variability rather than to a true lack of biological relevance.

Socioeconomic and social determinants and sleep-related exposures showed more moderate or heterogeneous associations. Neighborhood deprivation [80], socioeconomic position [79], and composite social determinants of health [81] were positively linked to EAA, while sleep quality and patterns yielded mixed results [83,84,85]. These findings further highlight the importance of structural and social factors within the broader exposome.

The 11 studies retained only for narrative synthesis provide additional valuable insights. They address heterogeneous exposures, including mixed lifestyle profiles [26,27], smoking across life stages [28,29,30], cognitive function [31], reproductive aging [35], chronic disease states [33,34], and specific prenatal or environmental toxicants [32,36]. Although not suitable for quantitative pooling, these studies enrich the overall picture and point to emerging frontiers in life-course and disease-specific epigenetic research.

#### Population- and Ancestry-Related Variability in Epigenetic Aging

Although the present meta-analysis integrates studies from diverse geographic regions, population-specific factors likely contribute to variability in epigenetic aging trajectories. Genetic background influences DNA methylation architecture, including ancestral-related differences in CpG methylation patterns and allele frequencies at methylation quantitative trait loci (meQTLs), which may modulate the sensitivity of epigenetic clocks to environmental and behavioral exposures [113]. Beyond genetic variation, lifestyle, environmental conditions, and socioeconomic structures differ substantially across populations and may interact with biological pathways underlying EAA [114]. For example, disparities in air pollution exposure, dietary patterns, psychosocial stress burden, and access to health-promoting resources can shape epigenetic aging through inflammatory, neuroendocrine, and metabolic mechanisms.

These considerations help contextualize the heterogeneity observed across studies and underscore that EAA reflects the combined influence of both inherited and exposome-related factors. Importantly, the consistency of associations across populations in the present synthesis suggests that, despite baseline differences in epigenetic profiles, the directionality of modifiable determinants is broadly conserved [115]. Nevertheless, future research should prioritize population-stratified analyses and the development of ancestry-informed epigenetic aging models to improve precision and generalizability across global populations.

### 4.2. Consistency Across Analytical Pools

Findings from secondary analytical pools (B, C, and D) reinforce and substantially extend the primary results obtained in Pool A, providing multi-metric validation of the observed associations. This convergence across different statistical frameworks is a major strength of the present synthesis, as it mitigates concerns related to scale-dependent or metric-specific biases and enhances the robustness and generalizability of the conclusions.

In Pool B (standardized effect sizes), consistent associations were observed across a wide range of exposures. Psychosocial factors such as aging anxiety [88], behavioral traits including self-control [90], and social relationships [92] showed significant standardized effects, mirroring the patterns seen in the primary analysis. Lifestyle factors, including physical activity [89] and various dietary patterns [93], also demonstrated protective standardized associations. The inclusion of evidence from randomized intervention settings in this pool is particularly noteworthy. Combined supplementation and exercise interventions [91] produced measurable improvements in epigenetic aging trajectories, offering preliminary support for causal plausibility and highlighting the potential reversibility of accelerated epigenetic aging through targeted lifestyle modifications.

Pool C (odds ratios) further confirmed an increased likelihood of accelerated epigenetic aging in relation to several modifiable exposures. Tobacco use [98,101], environmental toxicants [97], adverse dietary patterns [100,104], metabolic dysfunction [102,105], and sleep traits evaluated through genetic instruments in Mendelian randomization designs [103] were all associated with higher odds of accelerated EAA. The use of Mendelian randomization approaches in some of these studies is especially valuable, as it helps address residual confounding and strengthens causal inference for selected exposures.

Pool D (hazard ratios) extended the findings to clinically meaningful longitudinal outcomes. Associations were observed between environmental characteristics such as green and blue space availability [106], favorable dietary patterns [107], cardiorespiratory fitness [108], and lower inflammatory burden [109] with reduced hazard of accelerated aging phenotypes over time. These results are particularly relevant because they demonstrate that the exposures associated with EAA in cross-sectional and short-term studies also translate into differences in long-term biological aging trajectories, reinforcing the prognostic value of epigenetic clocks.

The overall convergence of results across all four analytical pools—unstandardized betas (Pool A), standardized effects (Pool B), odds ratios (Pool C), and hazard ratios (Pool D)—substantially reduces concerns regarding metric-specific bias and provides a high degree of triangulation. This multi-metric consistency strengthens confidence in the main findings and supports their generalizability across different statistical representations and study designs. Moreover, the inclusion of both observational and interventional evidence, as well as genetic instrumental variable approaches, adds complementary layers of support for the modifiability of epigenetic aging and the biological plausibility of the observed associations.

### 4.3. Integration with Exceptional Longevity and Blue Zone Research

The molecular findings of this meta-analysis show remarkable consistency with epidemiological observations from populations characterized by exceptional longevity. The Cilento region in southern Italy, identified as a candidate Blue Zone, is characterized by a unique combination of traditional Mediterranean dietary patterns, sustained physical activity integrated into daily life, favorable environmental conditions (including mild climate and high-quality air and water), and strong intergenerational social cohesion. Previous work conducted by part of the present research group (Aliberti et al., 2023; Aliberti et al., 2025a, 2025b, 2025c, 2025d) [109,110,111,112,116] has extensively characterized this exposome and documented exceptionally high rates of healthy longevity and successful aging in this area.

The present meta-analysis provides a critical translational bridge by demonstrating at the epigenetic level that the same modifiable factors associated with exceptional longevity at the population level—low exposure to air pollution and environmental toxicants, favorable metabolic and inflammatory profiles, reduced psychosocial stress, high diet quality, regular physical activity, and supportive social environments—are indeed linked to slower epigenetic aging. This convergence between macro-level epidemiological patterns observed in Cilento and other Italian longevity hotspots (including Sicilian Mountain villages) and micro-level epigenetic evidence strengthens the plausibility of a shared mechanistic framework. It suggests that the exposome of these long-lived populations operates, at least partly, by preserving epigenetic stability and slowing the biological aging process. Such integration moves the field forward from descriptive observations of longevity to a more mechanistic understanding of how specific modifiable determinants influence healthspan at the molecular level.

### 4.4. Novelty and Contribution to the Literature

A major contribution of this work is the development of the Modifiable Epigenetic Aging Burden Index (MEAB-Index). By synthesizing category-specific pooled estimates through inverse-variance weighting and bootstrap validation, the MEAB-Index moves beyond isolated associations toward a cumulative, prevention-oriented quantification of the modifiable burden on biological aging. To our knowledge, this represents the first meta-analytic framework that systematically integrates multiple exposure domains, harmonizes heterogeneous effect measures across four analytical pools, and operationalizes the cumulative impact of modifiable determinants on EAA.

The multi-pool analytical strategy, combined with rigorous sensitivity analyses, leave-one-out procedures, and assessment of publication bias, addresses several limitations common in previous reviews in the geroscience field. Furthermore, the explicit separation of studies suitable for quantitative synthesis from those retained for narrative review provides transparency regarding the current state of evidence standardization. These methodological advancements offer a reproducible template for future updates of this rapidly evolving literature and for comparative analyses across different populations and epigenetic clocks.

### 4.5. Heterogeneity and Meta-Regression

As expected, given the breadth of exposures, populations, and epigenetic clocks included, substantial between-study heterogeneity was observed. Meta-regression analyses identified exposure category and type of epigenetic clock as significant moderators of this variability, while study design and level of covariate adjustment showed more limited influence. Heterogeneity was particularly pronounced in environmental and metabolic domains, likely reflecting differences in exposure assessment (e.g., specific pollutants versus composite indices), timing of exposure across the life course, and population susceptibility. In contrast, psychosocial stress and socioeconomic domains showed relatively lower dispersion, suggesting more consistent underlying biological pathways.

Importantly, the direction and statistical significance of associations remained highly consistent across subgroups and sensitivity analyses. This pattern indicates heterogeneity primarily reflects differences in effect magnitude rather than conflicting evidence. Such variability is inherent to the multidimensional and context-dependent nature of biological aging and should be viewed as informative rather than problematic, highlighting the need for greater standardization in future studies while simultaneously revealing the richness of exposome-biology interactions.

### 4.6. Robustness and Bias Assessment

The robustness of the findings was thoroughly evaluated through multiple sensitivity analyses. Restriction to fully adjusted models, exclusion of studies with high risk of bias, and leave-one-out analyses consistently confirmed the stability of the main results. Although formal tests (Egger’s and Begg’s) and visual inspection of funnel plots suggested possible asymmetry in some domains, the trim-and-fill method identified no missing studies, and the adjusted pooled estimates remained virtually unchanged. These findings collectively support the internal validity and stability of the meta-analytic conclusions.

#### Epigenetic Regulatory Pathways, Reversibility, and Conceptual Limitations of Epigenetic Clocks

Emerging evidence indicates that modifiable exposures influence epigenetic aging not only through systemic pathways such as inflammation, oxidative stress, and neuroendocrine activation, but also through direct regulation of DNA methylation machinery. Environmental and behavioral factors have been shown to alter the activity of DNA methyltransferases (DNMT1, DNMT3A/3B) and ten-eleven translocation (TET) dioxygenases, thereby modulating methylation and demethylation dynamics at specific CpG sites that contribute to epigenetic clock algorithms [117].

A growing body of research also suggests that epigenetic aging is at least partially reversible. Lifestyle interventions, dietary modifications, physical activity programs, and selected pharmacological or nutraceutical approaches have demonstrated short-term reductions in epigenetic age estimates, supporting the notion that biological aging trajectories can be modulated through behavioral and environmental change [114]. Although the durability of these effects remains uncertain, these findings highlight the importance of critical windows across the life course—particularly early development, adolescence, and midlife—during which interventions may exert stronger or more sustained impacts.

At the same time, several conceptual limitations of epigenetic clocks warrant consideration. Different clocks capture distinct biological domains and vary in predictive performance for health outcomes, reflecting differences in training datasets, CpG selection, and underlying biological assumptions [5]. Tissue-specific methylation patterns may limit the generalizability of blood-derived clocks, and epigenetic age does not fully capture the multidimensional nature of biological aging. Moreover, correlations between epigenetic age acceleration and actual physiological aging processes, while robust, are not perfect and may differ across populations, tissues, and environmental contexts. Recognizing these limitations is essential for interpreting the present findings and for guiding the development of next-generation, context-sensitive aging biomarkers.

### 4.7. Strengths and Limitations

This study has several strengths, including the large number of included studies, rigorous harmonization of heterogeneous effect measures, a pre-specified multi-pool analytical strategy, comprehensive sensitivity analyses, and the innovative construction of the MEAB-Index with bootstrap validation. The integration of narrative synthesis for studies not suitable for quantitative pooling further enriches the interpretative depth of the review.

Limitations must also be acknowledged. The predominance of observational (mostly cross-sectional) designs limits causal inference, and residual confounding or reverse causation cannot be entirely excluded. Variability in exposure measurement, timing, and epigenetic clock selection contributed to heterogeneity, although this was partially addressed through subgroup and meta-regression analyses.

The deliberate inclusion of cross-sectional studies was a methodological choice aimed at maximizing coverage of modifiable determinants, given the current scarcity of longitudinal and interventional studies with EAA as the primary outcome. While this approach provides a broad map of associations, it underscores the urgent need for more high-quality longitudinal and intervention studies in this field.

### 4.8. Critical Interpretation

While the consistency of associations across multiple analytical approaches and exposure domains supports the robustness of the findings, caution is warranted in their interpretation. The observational nature of most included studies precludes definitive causal claims, and bidirectional relationships between exposures and biological aging cannot be excluded. For example, metabolic dysfunction may both contribute to and result from accelerated epigenetic aging.

Nevertheless, the triangulation of evidence across observational studies, Mendelian randomization analyses, and emerging interventional trials, together with the strong biological plausibility of the identified pathways, substantially strengthens the interpretation that modifiable determinants are likely to exert directional influences on epigenetic aging trajectories.

## 5. Public Health, Clinical, and Preventive Medicine Implications

The present findings have important translational implications for preventive medicine, clinical practice, and public health policy. By demonstrating that epigenetic age acceleration is meaningfully modifiable through changes in environmental, behavioral, and social determinants, this meta-analysis strengthens the rationale for incorporating biological aging biomarkers into population health frameworks [4,5]. Rather than limiting their use to individual risk stratification, epigenetic clocks can serve as sensitive indicators of cumulative exposome burden, enabling a shift toward system-level and upstream prevention strategies. The newly developed Modifiable Epigenetic Aging Burden Index (MEAB-Index) offers a novel quantitative tool to estimate the potential public health gains achievable by reducing harmful exposures and to prioritize interventions according to their expected impact on healthy longevity.

From a public health perspective, these findings support a paradigm shift from disease-centered to aging-centered prevention [118,119]. Epigenetic age acceleration appears to function as an integrative marker of lifetime exposure to modifiable determinants, capturing the cumulative biological imprint of air quality, metabolic health, psychosocial stress, and social conditions. This positions EAA as a valuable population-level surveillance metric that could help identify vulnerable groups and evaluate the long-term effectiveness of structural interventions [120]. Policies aimed at improving environmental quality, promoting metabolic health, mitigating chronic stress, and reducing socioeconomic inequalities may therefore yield substantial benefits not only for specific diseases but for the overall pace of biological aging in populations [121,122,123].

From a preventive medicine standpoint, the findings underscore the importance of early and sustained intervention across the entire life course. The associations observed suggest that biological aging trajectories are shaped long before the clinical manifestation of age-related diseases, reinforcing the value of integrated strategies spanning primary, secondary, and tertiary prevention [124,125]. Lifestyle modifications targeting metabolic and inflammatory pathways, reduction of psychosocial stressors, and optimization of environmental and social conditions represent high-yield opportunities for slowing epigenetic aging and extending healthspan.

In the clinical setting, epigenetic biomarkers have the potential to serve as dynamic, intermediate endpoints for monitoring individual responses to lifestyle or pharmacological interventions. Serial measurement of EAA could help personalize prevention plans and assess the efficacy of multifaceted programs [126]. However, the greatest public health impact is likely to occur at the population level, where structural interventions—such as urban planning to increase green spaces [127], policies to reduce air pollution [128], community programs to alleviate psychosocial stress [129], and initiatives to improve social cohesion—may produce the most meaningful and equitable benefits.

These implications are highly consistent with a One Health framework, which recognizes biological aging as the result of interconnected environmental, social, and behavioral systems. Interventions targeting urban design, environmental quality, food systems, and social environments are likely to influence aging trajectories while simultaneously improving broader health outcomes. Framing biological aging as a preventable and policy-sensitive outcome provides a powerful metric for evaluating the long-term effectiveness of cross-sectoral public health strategies, linking health promotion with environmental sustainability and social equity agendas [130,131,132].

## 6. Conclusions

This systematic review and meta-analysis provides robust evidence that epigenetic age acceleration is associated with a broad spectrum of modifiable behavioral, environmental, and social determinants. Taken together, these findings support the view that biological aging is a dynamic process influenced by upstream exposome factors across the life course.

The development of the MEAB-Index offers an initial, scalable framework for quantifying the potentially preventable component of epigenetic aging and illustrates how heterogeneous evidence can be integrated into a coherent, prevention-oriented metric. The multi-pool analytical strategy and extensive sensitivity analyses strengthen the reliability and interpretability of the results.

While most included studies were observational, the consistency of associations across diverse analytical approaches, exposure domains, and study designs suggests that modifiable determinants exert meaningful influences on epigenetic aging trajectories. Future research would benefit from longitudinal and interventional designs with repeated epigenetic assessments, as well as greater standardization of exposure measurement.

Overall, this work highlights the potential value of incorporating biological aging measures into preventive strategies aimed at improving healthspan and reducing health inequalities. Translating these molecular insights into public health and clinical practice may contribute to promoting healthier longevity at both individual and population levels.

## Figures and Tables

**Table 1:** Characteristics of included studies (*n* = 83) stratified by analytical pool.

Characteristic	Overall (*n* = 83)	Pool A (*n* = 60)	Pool B (*n* = 9)	Pool C (*n* = 10)	Pool D (*n* = 4)
**Study design**					
Cross-sectional	42 (50.6%)	28 (46.7%)	5 (55.6%)	7 (70.0%)	2 (50.0%)
Prospective cohort	29 (34.9%)	22 (36.7%)	3 (33.3%)	2 (20.0%)	2 (50.0%)
Other (longitudinal, nested, etc.)	12 (14.5%)	10 (16.7%)	1 (11.1%)	1 (10.0%)	0
**Region**					
USA/North America	48 (57.8%)	31 (51.7%)	6 (66.7%)	8 (80.0%	3 (75.0%)
Europe	21 (25.3%)	18 (30%)	2 (22.2%)	1 (10.0%)	0
Asia/Other	14 (16.9%)	11 (18.3%)	1 (11.1%)	1 (10.0%)	1 (25.0%)
**Exposure Category**					
Lifestyle/Behavioral	35 (42.2%)	24 (40.0%)	5 (55.6%)	4 (40.0%)	2 (50.0%)
Environmental	19 (22.9%)	15 (25.0%)	2 (22.2%)	2 (20.0%)	0
Socioeconomic/Psychosocial	16 (19.3%)	12 (20.0%)	1 (11.1%)	2 (20.0%)	1 (25.0%)
Mixed	13 (15.7%)	9 (15.0%)	1 (11.1%)	2 (20.0%)	1 (25.0%)
**Main Clocks**					
GrimAge/GrimAge2	26 (31.3%)	19 (31.7%)	3 (33.3%)	3 (3.30%)	1 (25.0%)
PhenoAge	21 (25.3%)	14 (23.3%)	3 (33.3%)	3 (30.0%)	1 (25.0%)
Horvath	14 (16.9%)	11 (18.3%)	1 (11.1%)	1 (10.0%)	1 (25.0%)
Multiple	15 (18.1%)	12 (20.0%)	2 (22.2%)	1 (10.0%)	0
**Adjustment**					
Full adjusted	66 (79.5%)	48 (80.0%)	7 (77.8%)	8 (80.0%)	3 (75.0%)
Minimally/Unadjusted	17 (20.5%)	12 (20.0%)	2 (22.2%)	2 (20.0%)	1 (25.0%)
Mean Sample Size	12,847	8742	4856	28,340	31,200
Mean Age (years)	56.4	55.8	57.2	58.1	59.3

Note: Values are presented as *n* (%) unless otherwise specified. Sample size is reported as median (range). “Full adjusted” refers to models that included at least age, sex, and major confounders (e.g., smoking status, body mass index, and socioeconomic position). “Other” study designs include twin studies, Mendelian randomization, and repeated-measures longitudinal designs. Exposure categories are not mutually exclusive in studies evaluating mixed determinants. Percentages may not sum to 100 due to rounding. Pool A = primary analysis (unstandardized β); Pool B = sensitivity analysis (standardized β/SMD); Pools C and D = secondary analyses (log OR and log HR, respectively).

**Table 2:** Summary of pooled effect sizes according to analytical pools.

Pool	Effect Metric	*n*. Studies (Associations)	Pooled Estimate (95% CI)	Heterogeneity (I^2^)	τ^2^	*p*-Value	Interpretation
**A (Primary)**	Unstandardized β (years of EAA)	60 (82)	+0.310 (+0.255 to +0.366)	98.8	0.024	<0.001	Significant moderate acceleration
**B (Sensitivity)**	Standardized β/SMD (SD units)	9 (14)	+0.071 (−0.104 to +0.245)	99.3	0.069	0.427	Non-significant; CI crosses zero
**C**	Odds Ratio (log-transformed)	10 (12)	1.749 (1.348 to 2.269)	95.7	0.144	<0.001	Significant increased odds
**D**	Hazard Ratio (log-transformed)	4 (10)	0.827 (0.639 to 1.069)	98.9	0.065	0.146	Non-significant; limited power

Note: Random-effects meta-analysis using the DerSimonian–Laird estimator. β values in Pool A represent years of epigenetic age acceleration per unit increase in adverse exposure. For Pools C and D, estimates are presented on the original scale (OR and HR) with log-transformed values used for pooling. Heterogeneity was quantified with I^2^ and τ^2^. All analyses were pre-specified. k = number of studies.

**Table 3:** Pooled estimates from random-effects models by exposure category (Pool A, *n* = 60 studies).

Exposure Category	k	Pooled β	95% CI Lower	95% CI Upper	*p*-Value	I^2^	Exposure Unit Definition and Measurement
**OVERALL (all 60 studies)**	60	+0.310	+0.255	+0.366	<0.001	98.8%	Composite across all categories. Individual study-level exposures were reported on heterogeneous scales (per 1-unit, per SD, per IQR, or categorical contrasts) and harmonized using pre-specified conversion rules.
**Environmental exposure**	9	+0.466	+0.167	+0.765	0.002	96.9%	Per IQR or per unit increase in ambient pollutant concentration (PM_2.5_, PM_10_, NO_2_ in µg/m^3^), per SD increase in chemical mixture exposure (PAH clusters, pesticide indices), per IQR increase in temperature variability (°C).
**Metabolic/Inflammatory**	6	+0.913	+0.468	+1.358	<0.001	99.4%	Per unit increase in composite metabolic or inflammatory index: cardiometabolic index (ln-CMI), metabolic syndrome severity score (0–5), systemic immune-inflammation index (SII, per 50-unit), fatty liver index (FLI, per unit increase).
**Psychosocial stress**	8	+0.188	+0.071	+0.304	0.002	85.3%	Per unit increase in validated psychometric score (ACEs count, BDI-II per 10 points, stressful life events inventory), per category of job strain (effort–reward imbalance).
**Socioeconomic/Social**	4	+0.174	−0.056	+0.405	0.139	77.9%	Per category or percentile increase in Area Deprivation Index (ADI), per level of upward/downward social mobility (childhood vs. adulthood social class)
**Sleep**	3	+0.006	−0.147	+0.160	0.936	84.3%	Per unit increase in Pittsburgh Sleep Quality Index (PSQI) global score, per unit increase in apnea-hypopnea index (AHI, events/hour).
**Diet & Nutrition**	12	−0.063	−0.323	+0.197	0.637	98.6%	Per SD or per interquintile increase in diet quality score (HEI-2020, AHEI, MDS, EAT-Lancet index), per drink/day of alcohol, per doubling of dietary fatty acid intake (µmol/L).
**Physical activity**	7	−0.278	−0.965	+0.408	0.427	95.2%	Per SD increase in objectively measured physical activity (steps/day, MET-hours, % time in MVPA via accelerometry), per category of leisure-time physical activity (active vs. inactive twin pairs)

Note: Results are from random-effects meta-analyses (DerSimonian–Laird estimator). β coefficients represent the change in epigenetic age acceleration (years) per unit increase in the adverse exposure (or per unit improvement for protective factors). k = number of studies included in each subgroup. 95% CI = 95% confidence interval. I^2^ = percentage of total variation due to heterogeneity. Categories with k < 3 were retained only for narrative synthesis and therefore were not included in this table. All analyses and harmonization procedures were pre-specified.

**Table 4:** MEAB-Index summary metrics.

Metric	β (Years)	SE	Bootstrap 95% CI	Input	Use
**MEAB-composite**	+0.153	0.039	+0.077 to +0.230	All 7 categories	Comprehensive
**MEAB-positive**	+0.263	0.054	+0.158 to +0.368	3 significant	Conservative
**CPB**	+1.566	0.280	+1.011 to +2.123	3 significant (sum)	Preventive estimate

Note: All metrics were derived using inverse-variance weighting and validated with 10,000 bootstrap iterations (percentile method).

## Data Availability

The data that support the findings of this study are derived from publicly available sources cited in the article. The dataset generated during the current study is available from the corresponding author upon reasonable request. To enhance transparency and reproducibility, all analysis scripts, harmonized datasets, and reproducible materials used in this meta-analysis are openly accessible in the dedicated GitHub repository: https://github.com/smaliberti/eaa-meta-analysis (GitHub repository) (accessed on 28 May 2026).

## Supplementary Materials

The following supporting information can be downloaded at: https://www.mdpi.com/article/10.3390/ijms27115032/s1.

## Abbreviations

The following abbreviations are used in this manuscript:

BMI	Body Mass Index
CI	Confidence Interval
CPB	Cumulative Preventable Burden
CpG	Cytosine-phosphate-Guanine
DL	DerSimonian–Laird
DNA	Deoxyribonucleic Acid
EAA	Epigenetic Age Acceleration
GRADE	Grading of Recommendations, Assessment, Development and Evaluation
HR	Hazard Ratio
I^2^	I-squared (heterogeneity statistic)
MEAB-Index	Modifiable Epigenetic Aging Burden Index
MEDLINE	Medical Literature Analysis and Retrieval System Online
NOS	Newcastle–Ottawa Scale
OR	Odds Ratio
PICOS	Population, Exposure, Comparator, Outcome, Study design
PMC	PubMed Central
PRISMA	Preferred Reporting Items for Systematic Reviews and Meta-Analyses
PROSPERO	International Prospective Register of Systematic Reviews
RoB 2	Cochrane Risk of Bias 2 tool
SD	Standard Deviation
SE	Standard Error
SMD	Standardized Mean Difference
τ^2^	Tau-squared (between-study variance)
