# Supplementary material for: Epigenetic Age Acceleration as a Modifiable Public Health Target: A Systematic Review and Meta-Analysis of Environmental, Behavioral, and Social Determinants with Development of the MEAB-Index
Source: Int J Mol Sci. 2026 Jun 2;27(11):5032. doi: 10.3390/ijms27115032 (PMC13256709; doi:10.3390/ijms27115032)
Supplement: Supplementary file 1 [file ijms-27-05032-s001.zip › Supplementary Table S1. ΓÇö Pool A.pdf]

Supplementary Table S1. Detailed results of Pool A (primary analysis): unstandardised beta coefficients or mean differences in epigenetic age acceleration (n = 60 studies)

Study ID	First Author	Year	Country	Study Design	Sample Size	Mean Age	Exposure Category	Specific Exposure	Clock Type	EAA Metric	Effect Type	Effect Value	CI Lower	CI Upper	SE	yi	sei	Adjustment Level	Main Covariates	Direction	Notes
1-s2.0-S001393512502537X	Yueli Yao	2026	Germany	cohort	1646	53.9	air pollution	PM2.5; PM10; PNC; PMcoarse; PM2.5abs; NO2; NOx; O3	DNAmHorvathAge ; DNAmHannumAge ; DNAmPhenoAge; ...	age acceleration (difference between epigenet)	absolute change in epigenetic age a...	0.120	+0.050	+0.190	0.0357	**+0.120**	0.0357	Multivariable	age, sex, white blood cell proportions (monocytes, natural killer cells, CD4+ T cells, CD8+ T cells.	**Positive**	Years per IQR; note IQR-scaled exposure
1-s2.0-S001393512502626 6	Zuo	2026	Netherlands	cross-sectional	1622	46.0	occupational pesticide exposur...	general pesticides; herbicides; insecticides; fung...	Horvath; Hannum; Skin & Blood; PhenoAge; Grim...	epigenetic age acceleration residuals	beta coefficient (years of age acce...	1.150	+0.180	2.110	0.4923	**1.150**	0.4923	Multivariable	age, sex, education level, income, smoking status, co-exposure to dusts and solvents	**Positive**	Years; primary metric
1-s2.0-S016041202200428 7	Baranyi	2022	United Kingdom	Longitudinal cohort study	525	72.5	Air pollution	Composite air quality index (AQI) based on PM2.5, ...	Horvath DNAmAge, Hannum DNAmAge, DNAm PhenoAg...	Epigenetic age acceleration (years) and DNAm	Regression coefficient (beta)	0.322	+0.088	+0.555	0.1191	**+0.322**	0.1191	Multivariable	Age, sex, parental occupational social class, childhood smoking, years spent in education	**Positive**	Linear regression beta
1-s2.0-S053155652500346 8	Hye-Young Park	2026	South Korea	Longitudinal cohort study	1452	59.7	Metabolic syndrome severity	MetS severity score (0-5 based on diagnostic crite...	PhenoAge, GrimAge2, DunedinPACE	Epigenetic age acceleration residuals	Beta coefficient	0.250	+0.040	+0.470	0.1097	**+0.250**	0.1097	Multivariable	Chronological age, sex, education, smoking status, drinking status, physical activity, BMI, income	**Positive**	Linear regression beta
1-s2.0-S127977072600007 2	Xinyu Zhang	2026	United States	cross-sectional	15050	51.0	vitamin intake	mixture of 11 dietary vitamins (A, B1, B2, B3, B6,...	Klemera-Doubal method biological age (KDM-BA)...	KDM-acceleration; PhenoAge-acceleration; HD	beta coefficient (linear regression...	-1.281	-2.033	-0.530	0.3834	**-1.281**	0.3834	Multivariable	age, sex, race, educational level, marital status, poverty income ratio, BMI, smoking status, alcohol	**Negative**	Linear beta
1-s2.0-S127977072600009 6	Kai Wei	2026	United States	cross-sectional	12806	—	marijuana use	current marijuana use (past 30 days) vs never use	PhenoAge; KD-BioAge	residuals from linear regression of biological	regression coefficient (β)	0.720	+0.410	1.020	0.1556	**+0.720**	0.1556	Multivariable	age, sex, race/ethnicity, marital status, education, family income, smoking, alcohol consumption	**Positive**	Linear beta
1-s2.0-S258900422502644 6	Yu	2026	Sweden	longitudinal repeated-mea...	807	25.9	ambient temperature	short-term ambient temperature exposure (daily ind...	inflammation-related proteomic aging clock	proteomic age acceleration	mean difference	0.050	+0.030	+0.100	0.0179	**+0.050**	0.0179	Multivariable	age, sex, long time trend, phases, day of week, season, SARS-CoV-2 infection history, vaccination.	**Positive**	Mean difference — assume years given EAA context

Supplementary Table S1. Detailed results of Pool A (primary analysis): unstandardised beta coefficients or mean differences in epigenetic age acceleration (n = 60 studies)

Study ID	First Author	Year	Country	Study Design	Sample Size	Mean Age	Exposure Category	Specific Exposure	Clock Type	EAA Metric	Effect Type	Effect Value	CI Lower	CI Upper	SE	yi	sei	Adjustment Level	Main Covariates	Direction	Notes
10522_2025_Article_10195	Wu	2025	Taiwan	cross-sectional	2474	49.8	Lifestyle factors; physiologic...	Smoking; BMI; waist-hip ratio; hemoglobin A1c; HDL...	GrimAge (version 2)	GrimEAA (AgeAccelGrim 2)	regression coefficient (beta)	5.176	4.798	5.553	0.1923	**5.176**	0.1923	Multivariable	Sex, BMI, waist-hip ratio, smoking status, hemoglobin A1c, HDL-C, creatinine, uric acid, gamma-gluta.	**Positive**	Linear beta
13148_2019	Sillanpää	2019	Finland	Twin study with quantitat...	1249	—	Leisure-time physical activity	Long-term leisure-time physical activity discordan...	Horvath's DNA methylation age	Age acceleration residuals from linear regres...	Mean difference in DNAm age acceleration	-1.170	-3.430	1.100	11.556	**-1.170**	11.556	Multivariable	Sex, smoking (never, former, current), BMI	**Negative**	Twin-design mean difference in years
13148_2024_Article_1706	Lee	2024	South Korea	cohort	692	54.0	sleep quality	Pittsburgh Sleep Quality Index (PSQI) global score	GrimAgeAccel; DunedinPACE	epigenetic age acceleration	beta coefficient	0.167	+0.038	+0.296	0.0660	**+0.167**	0.0660	Multivariable	chronological age, sex, smoking status, drinking status, BMI	**Positive**	Linear beta
41598_2022_Article_26433	Bao	2022	United Kingdom	Cross-sectional analysis ...	3140	54.5	Socioeconomic position and soc...	Childhood social class, adulthood social class, so...	Horvath; Hannum; Phenoage; DunedinPoAm	Residuals of linear regression of DNAm age on...	Mean difference in age acceleration	0.470	+0.190	+0.750	0.1429	**+0.470**	0.1429	Multivariable	Sex, age, age squared, batch effect (plate), white blood cell composition; sensitivity analyses	**Positive**	Mean difference in years
41598_2022_Article_8160	Cardenas	2022	Costa Rica	cross-sectional analysis ...	489	79.4	cigarette smoking	smoking status (current, former, never); ever smok...	Horvath Pan Tissue; Horvath Skin-Blood; Hannu...	AgeAccelerationResidual (residuals of epigene...	mean difference in years	3.070	2.410	3.740	0.3393	**3.070**	0.3393	Multivariable	sex, chronological age, BMI, education, household assets, genetic principal components (PC1, PC2)	**Positive**	Years EAA relative to non-smokers; DNAmTL kb part — note in ...
ACEL-22-e13779	Joshi	2023	Canada	cross-sectional	1445	63.0	Adverse childhood experiences ...	Cumulative ACEs score; individual ACEs including e...	DNAm GrimAge; DNAm PhenoAge	Epigenetic age acceleration residuals (regres...	Regression coefficient (β)	0.070	+0.020	+0.110	0.0230	**+0.070**	0.0230	Multivariable	Sex, annual household income, number of poor health behaviors (smoking, physical activity, alcohol.	**Positive**	Linear regression beta
ACEL-22-e13828	Fox	2023	Germany	cross-sectional	3567	55.5	Physical activity	Step counts; MET-Hours; % time in moderate-to-vigo...	GrimAge (main), also Hannum, Horvath, PhenoAg...	Epigenetic Age Acceleration (GrimAge accelera...	Regression coefficient (β)	-0.380	-0.650	-0.120	0.1352	**-0.380**	0.1352	Multivariable	Age, age², sex, education, batch effect, cell proportions, smoking status, season	**Negative**	Linear term extracted; quadratic term — verify which was ext...
ACEL-24-e70182	Brooke	2025	Austria/Germany/USA/De...	Longitudinal observationa...	24	26.2	Physical activity	Strenuous exercise (before vs. after soccer game)	DNAmGrimAge2, DNAmFitAge, Skin & Blood Clock	Calibrated epigenetic age (GrimAge2 cal., Fit...	Mean difference in epigenetic age	-7.070	-10.320	-3.710	16.900	**-7.070**	16.900	Adjusted	Chronological age, timepoints (before, after game, rested), player ID, batch number	**Negative**	Mean difference in years

Supplementary Table S1. Detailed results of Pool A (primary analysis): unstandardised beta coefficients or mean differences in epigenetic age acceleration (n = 60 studies)

Study ID	First Author	Year	Country	Study Design	Sample Size	Mean Age	Exposure Category	Specific Exposure	Clock Type	EAA Metric	Effect Type	Effect Value	CI Lower	CI Upper	SE	yi	sei	Adjustment Level	Main Covariates	Direction	Notes
aging-13-202814	Martin	2021	United States	cross-sectional analysis ...	158	54.0	Neighborhood social environmen...	Neighborhood poverty (z-score); Neighborhood quali...	Horvath; Hannum; Levine's PhenoAge	Age acceleration residuals (DNAm age regresse...	Beta coefficient (linear regression)	2.100	+0.400	3.800	0.8673	**2.100**	0.8673	Multivariable	Race/ethnicity, education, employment, smoking status, alcohol intake, years in neighborhood;	**Positive**	Standard linear beta
aging-14-203872	Freni-Sterrantino	2022	Finland	Cohort study (Northern Fi...	604	46.0	Work-related stress and employ...	Job strain, effort-reward imbalance, job status, w...	Horvath, Hannum, PhenoAge, GrimAge, DunedinPo...	Epigenetic age acceleration (years) and pace ...	Regression coefficient (β)	2.058	+0.517	3.599	0.7862	**2.058**	0.7862	Multivariable	Sex, education, smoking habit, alcohol consumption, BMI, physical activity	**Positive**	Years EAA; primary metric
aging-14-204327	Freni-Sterrantino	2022	United Kingdom	cross-sectional	631	—	Job stability	Unemployed vs paid employment	Horvath, Hannum, PhenoAge, GrimAge, DunedinPo...	Epigenetic age acceleration (years) and pace ...	Difference in epigenetic age acceleration	3.210	+0.890	5.530	11.837	**3.210**	11.837	Multivariable	sex, alcohol consumption, smoking, BMI, educational level	**Positive**	Years difference; DunedinPACE pace-of-aging part is dimensio...
aging-15-205153	Wang	2023	United States	cohort	3823	—	alcohol consumption	long-term average total alcohol consumption and ty...	GrimAge, PhenoAge, Horvath's age, Hannum's ag...	GrimAge acceleration (GAA), PhenoAge accelera...	beta coefficient	0.430	+0.136	+0.724	0.1500	**+0.430**	0.1500	Multivariable	sex, physical activity index, education level, BMI, smoking (pack-year), chronological age, lab inde...	**Positive**	Years per drink/day; primary metric
Bozack_2023_ClinicalEpigenetic...	Bozack	2023	—	pre-birth cohort	485	—	maternal smoking during pregna...	maternal smoking during pregnancy (vs. never smoke...	skin & blood clock	epigenetic age acceleration (EAA) at birth	beta coefficient (weeks)	1.170	-0.090	2.420	0.6403	**1.170**	0.6403	Multivariable	maternal age, pre-pregnancy BMI, education, prenatal smoking, newborn sex, preterm birth, birth weig...	**Positive**	CAUTION: unit is weeks not years; conversion_notes set; conv...
Bozack_2026	Anne K. Bozack	2026	United States	cross-sectional	1771	64.8	Dietary fatty acids	Total fatty acids; saturated fatty acids (SFA); mo...	Horvath1; Horvath2; Hannum; Lin; Zhang; Vidal...	Epigenetic age acceleration (years or SDs dep...	Beta coefficient	-1.050	-1.870	-0.220	0.4209	**-1.050**	0.4209	Multivariable	Age, age2, sex, race/ethnicity, BMI, education,	**Negative**	Years per doubling;
Chen_2024_Article_1707	Chen	2024	UK (data from UK Bioba...	Mendelian randomization	5E+05	54.8	Walking and sedentary behavior	Usual walking pace; walking duration; walking freq...	GrimAge; PhenoAge; Horvath; Hannum	Epigenetic age acceleration (EAA) residuals f...	Beta coefficient (IVW MR estimate)	-1.842	-2.937	-0.747	0.5587	**-1.842**	0.5587	Adjusted	Not explicitly reported; genetic instrumental variables selected to avoid confounding	**Negative**	Mendelian Randomisation IVW; causal beta estimate; flag MR f...

Supplementary Table S1. Detailed results of Pool A (primary analysis): unstandardised beta coefficients or mean differences in epigenetic age acceleration (n = 60 studies)

Study ID	First Author	Year	Country	Study Design	Sample Size	Mean Age	Exposure Category	Specific Exposure	Clock Type	EAA Metric	Effect Type	Effect Value	CI Lower	CI Upper	SE	yi	sei	Adjustment Level	Main Covariates	Direction	Notes
Diao_2025_CE	Tingyue Diao	2025	China	Cross-sectional and prosp...	3566	65.5	Sleep patterns	Sleep score integrating bedtime, sleep duration, s...	PhenoAgeAccel; GrimAgeAccel; DunedinPACE; DNA...	Epigenetic age acceleration residuals or dire...	Beta coefficient (linear regression)	-0.208	-0.369	-0.047	0.0821	**-0.208**	0.0821	Multivariable	Chronological age, sex, education level, smoking status, drinking status, physical activity	**Negative**	Standard linear beta
aging-16-205943	Chen	2024	United States	cohort	359	49.3	cognitive function	adolescent crystallized intelligence (PPVT-15); mi...	Horvath; Hannum; GrimAge; PhenoAge; DunedinPA...	residuals of epigenetic clocks regressed on c...	beta coefficient	-0.048	-0.077	-0.019	0.0150	**-0.048**	0.0150	Multivariable	adult socioeconomic status, sex, race/ethnicity, BMI, smoking, alcohol use, cell type proportions	**Negative**	Linear beta
EATLancet-2026-npjAging	Jie Li	2026	United Kingdom	cohort	87282	56.1	dietary patterns	EAT-Lancet diet index; plant-based diet index (PDI...	Klemera-Doubal biological age (KDM-BA); Pheno...	KDM-BA acceleration; PhenoAge acceleration; t...	beta coefficient (years or % change)	-0.530	-0.570	-0.490	0.0204	**-0.530**	0.0204	Multivariable	age, sex, race, Townsend deprivation index, BMI, smoking status, healthy drinking, physical activity.	**Negative**	Years part extracted; verify in conversion_notes
ebiom2019	Xiaoyu Li	2019	United States	cohort	622	68.7	Sleep disordered breathing	Apnea-hypopnea index (AHI); Percentage of sleep ti...	DNAm-PhenoAge (Levine's clock); DNAm-Age (Hor...	DNAm-PhenoAge acceleration; DNAm-Age accelera...	linear regression coefficient (beta)	0.030	+0.001	+0.060	0.0151	**+0.030**	0.0151	Multivariable	chronological age, sex, race/ethnicity, household income, study site, BMI, smoking status, drinking	**Positive**	Linear beta
gbae176	Eun Young Choi	2024	United States	Cross-sectional analysis ...	3146	68.8	Neighborhood social stressors	Socioeconomic deprivation; observed disorder; perc...	PCHorvath; PCHannum; PCPhenoAge; PCGrimAge; D...	Epigenetic age acceleration residuals (PC clo...	Regression coefficient (B)	0.022	+0.016	+0.028	0.0031	**+0.022**	0.0031	Multivariable	Age, gender, race/ethnicity, PLQ interview year, years in neighborhood, marital status	**Positive**	Unstandardized regression coefficient
glab149	Kresovich	2021	United States	prospective cohort (case-...	2316	55.3	Alcohol consumption	Average lifetime alcohol use (drinks/year drinking...	Hannum, Horvath, PhenoAge, GrimAge	Hannum AgeAccel, Horvath AgeAccel, PhenoAgeAc...	beta coefficient (years increase)	0.270	+0.040	+0.500	0.1173	**+0.270**	0.1173	Multivariable	education level (high school/GED, attended college, advanced degree), body mass index (continuous)	**Positive**	Years per alcohol unit; primary metric
glad118	Joshi	2024	Canada	cross-sectional	1445	59.7	Neighborhood deprivation; depr...	Neighborhood material and/or social deprivation (h...	DNAm GrimAge; DNAm PhenoAge	residuals from regression of biological age o...	regression coefficient (b)	0.660	+0.210	1.120	0.2321	**+0.660**	0.2321	Multivariable	sex, annual household income, number of people in household, number of poor health behaviors, number	**Positive**	Unstandardized b

Supplementary Table S1. Detailed results of Pool A (primary analysis): unstandardised beta coefficients or mean differences in epigenetic age acceleration (n = 60 studies)

Study ID	First Author	Year	Country	Study Design	Sample Size	Mean Age	Exposure Category	Specific Exposure	Clock Type	EAA Metric	Effect Type	Effect Value	CI Lower	CI Upper	SE	yi	sei	Adjustment Level	Main Covariates	Direction	Notes
harris_2024_oi_240863_17234703...	Harris	2024	United States	cohort	4237	38.4	Sociodemographic and lifestyle...	Education (no college vs college or higher), annua...	Second- and third-generation epigenetic clock...	Epigenetic age acceleration (years for PhenoA...	Beta coefficient (difference in year)	1.700	+0.680	2.720	0.5204	**1.700**	0.5204	Multivariable	Age, sex, race/ethnicity, immigrant generation, education, income, region, rural/urban residence	**Positive**	Reported in years; SD-unit cases need sub-flag — check effec...
ijms-26-01478	Choi	2025	United States	cross-sectional	589	63.1	Environmental chemical	Serum 2,3,7,8-Tetrachlorodibenzo-p-dioxin (TCDD) l...	Horvath Age; Hannum Age; SkinBlood Age; Pheno...	Epigenetic age acceleration (years)	Beta coefficient (years increase)	0.730	+0.122	1.338	0.3100	**+0.730**	0.3100	Multivariable	Age, sex, race/ethnicity, education, family income, smoking status	**Positive**	Years per TCDD unit; primary metric
JCSM-16-e13873	Ammous	2025	United States	Longitudinal cohort with ...	3873	68.9	Physical activity	Self-reported moderate-to-vigorous physical activi...	GrimAge, PhenoAge, DunedinPACE	Epigenetic age acceleration residuals for Gri...	Difference in epigenetic age accelation	-0.760	-1.090	-0.420	0.1709	**-0.760**	0.1709	Multivariable	Age, gender, race/ethnicity, educational attainment, total wealth, current smoker status, BMI	**Negative**	Years difference
kwaa251	Kresovich	2021	United States	cross-sectional	2758	57.0	body composition; physical act...	body mass index (BMI); waist-to-hip ratio (WtH); w...	Hannum; Horvath; PhenoAge; GrimAge	PhenoAge age acceleration; GrimAge age accele...	beta coefficient (years of epigenet.)	3.150	2.410	3.900	0.3801	**3.150**	0.3801	Multivariable	education, smoking status, alcohol intake, menopausal status, physical activity (for body composition)	**Positive**	Years; primary metric
lawrence_2020_oi_200797_160375...	Lawrence	2020	United States	cross-sectional	2630	56.9	Neighborhood deprivation	Area Deprivation Index (ADI) US percentile levels ...	Hannum; PhenoAge; GrimAge; Horvath	Epigenetic age acceleration residuals (z scor...	beta coefficient	0.230	+0.010	+0.450	0.1122	**+0.230**	0.1122	Multivariable	None (unadjusted weighted model); adjusted models included smoking status, environmental tobacco)	**Positive**	Linear beta
Liu_2025_NHANES_SII_EAA	Rundong Liu	2025	United States	Cohort study (NHANES 1999...	1950	63.7	Systemic immune-inflammation i...	SII = platelet count × (neutrophil count / lymphoc...	Horvath, Hannum, Skin & Blood, PhenoAge, Grim...	HorvathAccel, HannumAccel, Skin&BloodAccel, P...	Regression coefficient (β) per 50-u	0.213	+0.155	+0.270	0.0293	**+0.213**	0.0293	Multivariable	Age, gender, race, marital status, education level, poverty-to-income ratio, smoking status, alcohol	**Positive**	Years per 50-unit SII; note non-standard exposure unit
maunakea_2024_oi_240700_172133...	Maunakea	2024	United States	cohort study, cross-secti...	376	57.8	Neighborhood socioeconomic sta...	NSES (low vs high); Educational level (years compl...	Dunedin Pace of Aging Calculated From the Epi...	DunedinPACE score	beta coefficient	-0.005	-0.013	+0.002	0.0038	**-0.005**	0.0038	Adjusted	Age, sex, Healthy Eating Index, body mass index, neighborhood socioeconomic status	**Negative**	Linear beta

Supplementary Table S1. Detailed results of Pool A (primary analysis): unstandardised beta coefficients or mean differences in epigenetic age acceleration (n = 60 studies)

Study ID	First Author	Year	Country	Study Design	Sample Size	Mean Age	Exposure Category	Specific Exposure	Clock Type	EAA Metric	Effect Type	Effect Value	CI Lower	CI Upper	SE	yi	sei	Adjustment Level	Main Covariates	Direction	Notes
nihms-1695982	Stephenson	2021	Finland	Population-based cohort w...	1004	23.0	Alcohol consumption	Drinks per week	GrimAge	AgeAccelGrim	Regression coefficient (beta)	0.053	+0.035	+0.071	0.0090	**+0.053**	0.0090	Multivariable	Age, sex, BMI, smoking status, white blood cell composition	**Positive**	Linear regression beta
nihms-1818253	Liu	2022	United States	Co-twin control study (lo...	291	56.0	Depression	Beck Depression Inventory II (BDI-II) total score	HannumAA; EEAA; PhenoAA	DNA methylation age acceleration residuals	Difference in years of epigenetic	0.730	+0.130	1.330	0.3061	**+0.730**	0.3061	Multivariable	Zygosity, current smoking, coronary heart disease history, BMI, number of alcoholic drinks per week	**Positive**	Years per 10 BDI-II points; note exposure unit
nihms-1900975	Khodasevich	2023	United States	cohort	385	—	prenatal phthalate exposure	prenatal urinary concentrations of 11 phthalate me...	Bohlin's gestational age clock at birth; Horv...	Gestational Age Acceleration (GAA) at birth; ...	beta coefficient (linear regression)	-0.580	-1.020	-0.130	0.2270	**-0.580**	0.2270	Multivariable	maternal poverty category, parity, maternal age, maternal smoking, maternal BMI, child sex	**Negative**	Linear beta
nihms-1915286	Batel Blechter	2023	China	Observational cohort stud...	106	56.3	Household air pollution (HAP) ...	PAH clusters (PAH31, PAH33, PAH36), 5-methylchryse...	GrimAge	Epigenetic age acceleration (residuals of Gri...	Beta coefficient (years increase)	0.770	+0.360	1.190	0.2117	**+0.770**	0.2117	Multivariable	Chronological age, county, BMI, education, socioeconomic status; sensitivity analyses adjusted	**Positive**	Years per SD exposure; exposure unit is SD not raw — note in...
nihms-1937864	Wenli Ni	2023	Germany	longitudinal population-b...	2617	59.8	Air temperature	4-week and 8-week moving average air temperature; ...	HorvathAA; HannumAA; PhenoAA; GrimAA; SkinBlo...	Epigenetic age acceleration (difference betwe...	Change in epigenetic age acceleration	2.240	1.950	2.530	0.1480	**2.240**	0.1480	Multivariable	chronological age, sex, education, BMI, alcohol consumption, smoking status, physical activity	**Positive**	Mean difference / change score in years
nihms-1940244	Laubach	2024	United States	cohort	205	29.8	maternal social experiences	maternal experiences of racial bias or discriminat...	Horvath's Pan-Tissue clock; Horvath's Skin & ...	epigenetic age acceleration (residuals of epi...	mean difference in years of EAA	-0.690	-1.230	-0.130	0.2806	**-0.690**	0.2806	Multivariable	maternal prenatal smoking, child sex; Model 2 additionally adjusted for EAA at birth	**Negative**	Mean difference in years; primary metric
nihms-1943037	Koenigsberg	2023	United States	cross-sectional analysis ...	4126	—	Air pollution	PM2.5, PM10, NO2	DunedinPACE; GrimAgeAccel; PhenoAgeAccel; Hor...	GrimAgeAccel and DunedinPACE (primary); Pheno...	beta coefficient (years or unit change)	1.090	+0.160	2.030	0.4770	**1.090**	0.4770	Multivariable	DNA methylation array (450K, EPIC), chronological age, race/ethnicity (Black, NHW), education level	**Positive**	Years EAA; primary metric

Supplementary Table S1. Detailed results of Pool A (primary analysis): unstandardised beta coefficients or mean differences in epigenetic age acceleration (n = 60 studies)

Study ID	First Author	Year	Country	Study Design	Sample Size	Mean Age	Exposure Category	Specific Exposure	Clock Type	EAA Metric	Effect Type	Effect Value	CI Lower	CI Upper	SE	yi	sei	Adjustment Level	Main Covariates	Direction	Notes
nihms-2084935	Kurbanov	2025	Belgium	cross-sectional	16	70.0	Idiopathic Pulmonary Fibrosis ...	IPF disease status and severity groups (IPF1 early...	Horvath, Hannum, PhenoAge, GrimAge, GrimAge2,...	epigenetic age acceleration residuals from re...	mean difference in epigenetic age acceleration	4.100	+0.180	8.020	20.000	**4.100**	20.000	Adjusted	technical and biological factors correlated with principal components; smoking status included	**Positive**	Years / DunedinPACE rate; primary metric
s12889-024-21249-3	Xia	2025	United States	cross-sectional	3925	46.0	Sugar-sweetened beverage (SSB)...	Energy intake from SSB (kcal/day) categorized as n...	Phenotypic age (PhenoAge)	PhenoAgeAccel (phenotypic age acceleration)	beta coefficient (linear regression)	0.179	+0.086	+0.271	0.0472	**+0.179**	0.0472	Multivariable	gender, age, race/ethnicity, education level, marital status, poverty income ratio, smoking status	**Positive**	Linear beta
s12889-025-22053-3	Liu	2025	United States	cross-sectional	4282	50.0	Cardiometabolic index (CMI)	CMI (ln-transformed and quartiles)	biological age derived from 8 biomarkers (cli...	BioAgeAccel (biological age acceleration = bi...	beta coefficient (years increase)	1.160	1.020	1.310	0.0740	**1.160**	0.0740	Multivariable	age, sex, ethnicity, family PIR, education level, hypertension, diabetes, smoking status, alcohol.	**Positive**	Years per ln-unit CMI; primary metric
s12916-025-04480-6	Ohi	2025	Japan	case-control	296	35.0	anxiety disorders	anxiety disorder diagnosis (SAD, PD, GAD)	PCHannumG2013 (primary), ZhangQ2019, PCHorvat...	epigenetic age acceleration residuals (DNAm a...	regression coefficient (B)	-3.460	-5.734	-1.186	11.600	**-3.460**	11.600	Multivariable	sex, current smoking status, batch effects (slide variable)	**Negative**	Unstandardized B
s12979-025-00500-4	Lin	2025	USA; China	Observational; cross-sect...	12973	41.0	Psoriasis	Psoriasis status; Psoriasis Area and Severity Inde...	Phenotypic age (PhenoAge); Klemera-Doubal met...	PhenoAge advance (difference between biologic...	Adjusted beta coefficient	0.540	+0.120	+0.970	0.2168	**+0.540**	0.2168	Multivariable	Age, gender, race/ethnicity, education, income, physical activity, BMI, smoking, alcohol use, diabet.	**Positive**	Adjusted beta in years; use as-is
s13148-025-01827-x	Daredia	2025	United States	cross-sectional	658	66.1	Reproductive aging	Age at menopause	GrimAge	epigenetic age deviation (residuals from regr...	regression coefficient (B)	-0.100	-0.190	-0.020	0.0434	**-0.100**	0.0434	Multivariable	BMI, smoking status, alcohol intake, and estimated cell-type proportions considered	**Negative**	Unstandardized B
s40520-025-02964-2	Fei Shan	2025	United States	cross-sectional	3376	46.0	serum polyunsaturated fatty ac...	total n-6 PUFAs; linoleic acid (LA); gamma-linolen...	PhenoAge	PhenoAge Acceleration (PhenoAgeAccel)	beta coefficient (linear regression)	-0.508	-0.785	-0.232	0.1412	**-0.508**	0.1412	Multivariable	age, sex, race/ethnicity, education level, poverty-to-income ratio, body mass index, smoking status.	**Negative**	Linear beta

Supplementary Table S1. Detailed results of Pool A (primary analysis): unstandardised beta coefficients or mean differences in epigenetic age acceleration (n = 60 studies)

Study ID	First Author	Year	Country	Study Design	Sample Size	Mean Age	Exposure Category	Specific Exposure	Clock Type	EAA Metric	Effect Type	Effect Value	CI Lower	CI Upper	SE	yi	sei	Adjustment Level	Main Covariates	Direction	Notes
s41043-026-01255-4	Chapnick	2026	Guatemala	cross-sectional	1095	45.0	cardiometabolic conditions	obesity, diabetes, hypertension, metabolic syndrom...	DunedinPACE; PhenoAge; GrimAge	residuals from regressing epigenetic age on c...	beta coefficient	0.090	+0.070	+0.100	0.0077	**+0.090**	0.0077	Multivariable	sex, birth year, sibship clustering	**Positive**	Linear beta
s41467-025-67622-7	Jiang Li	2026	UK, US	cohort	3E+05	56.1	social determinants of health ...	combined SDH score (favorable, medium, unfavorable...	Klemera–Doubal biological age (KDM-BA); pheno...	KDM-BA acceleration; phenotypic age accelerat...	beta coefficient (years increase)	0.220	+0.210	+0.230	0.0051	**+0.220**	0.0051	Multivariable	age, sex, BMI, smoking status, alcohol drinking, diet, physical activity,	**Positive**	Years per SDH point; primary metric
s41538-025-00625-2	Xinming Xu	2025	United States	Cross-sectional and survi...	34330	46.7	Dietary patterns	Healthy Eating Index-2020 (HEI2020), Alternate Hea...	GrimAge2, DunedinPoAm, PhenoAge and organ-spe...	Acceleration of epigenetic and phenotypic age...	Beta coefficient (years change)	-1.850	-2.830	-0.870	0.5000	**-1.850**	0.5000	Multivariable	Age, sex, ethnicity, education, poverty-income ratio, smoking status, physical activity, BMI, diabet.	**Negative**	Years per quintile increment; consistent with primary metric
s41598-024-82747-3	Egorov	2025	United States	cross-sectional	116	57.7	residential greenness	distance-to-residence weighted tree cover, vegetat...	Hannum, Horvath, Levine PhenoAge, Li	residuals from linear regression of epigeneti...	change in EAA (years) per interquartil	-1.600	-2.600	-0.600	0.5102	**-1.600**	0.5102	Multivariable	race, sex, waist-to-hip ratio, height, smoking status, WBC types (CD4+ T cells, CD8+ T cells, granul.	**Negative**	Years per IQR; primary metric
sciadv.adl3747	Cui	2024	United Kingdom	population-based cohort s...	3E+05	56.4	early-life tobacco exposure	in utero tobacco exposure; age of smoking initiati...	Klemera-Doubal biological age (KDM-BA); Pheno...	KDM-BA acceleration; PhenoAge acceleration; t...	beta coefficient (years for KDM-BA	0.260	+0.240	+0.290	0.0128	**+0.260**	0.0128	Multivariable	age at recruitment, sex, ethnicity, birthplace	**Positive**	Years part extracted for PhenoAge/KDM; telomere % part flagg...
sex_differences_in_the_associa...	Yang	2025	United States	cross-sectional	16479	46.0	Metabolic dysfunction-associat...	Fatty liver index (FLI)	Phenotypic age (PhenoAge)	PhenoAge acceleration (PhenoAgeAccel)	beta coefficient and odds ratio	2.960	2.730	3.180	0.1148	**2.960**	0.1148	Multivariable	age, sex, race, education, marital status, poverty-income ratio, smoking status, drinking status, ph.	**Positive**	Extract beta for primary pool; OR part → pool C separately; ...
Skinner_2024_JAGS	Skinner	2024	United States	Cohort study (Women's Hea...	3988	—	Psychosocial stress	Stressful life events (SLEs) burden over past year...	GrimAge; DNAmTL	Epigenetic age acceleration (GrimAge accelera...	Regression coefficient (β)	0.340	+0.080	+0.590	0.1301	**+0.340**	0.1301	Multivariable	Income, education level, marriage status, BMI, genetic ancestry principal components, DNAm-estimated.	**Positive**	Linear regression beta

Supplementary Table S1. Detailed results of Pool A (primary analysis): unstandardised beta coefficients or mean differences in epigenetic age acceleration (n = 60 studies)

Study ID	First Author	Year	Country	Study Design	Sample Size	Mean Age	Exposure Category	Specific Exposure	Clock Type	EAA Metric	Effect Type	Effect Value	CI Lower	CI Upper	SE	yi	sei	Adjustment Level	Main Covariates	Direction	Notes
Suglia_2024	Suglia	2024	United States	cohort	359	50.0	psychosocial stressors	total life stress; childhood stress; adulthood str…	GrimAge; DunedinPACE; Skin & Blood; Horvath; …	age acceleration residuals (GrimAge, DunedinP)	beta coefficient	0.488	+0.182	+0.794	0.1560	**+0.488**	0.1560	Multivariable	sex, race, smoking status, cell composition (sensitivity analyses), batch effects (sensitivity analytics)	**Positive**	Linear beta
White_2019_EnvironInt	White	2019	United States	cross-sectional analysis …	2747	57.0	Air pollution	PM2.5, PM10, NO2; PM2.5 component clusters	Levine, Hannum, Horvath	Residuals of DNAm age regressed	beta coefficient per IQR increase i…	-0.240	-0.470	-0.020	0.1148	**-0.240**	0.1148	Multivariable	Future breast cancer case status, education (3 levels), smoking status (current, former, never).	**Negative**	Years per IQR; note IQR-scaled exposure
Xu_2026_Mediators_of_Inflammat…	Yangyu Xu	2026	United States	cross-sectional	1053	—	inflammatory marker	neutrophil percentage-to-albumin ratio (NPAR)	Klemera–Doubal method (KDM) and Phenotypic ag…	biological age acceleration	beta coefficient (linear regression…	0.860	+0.390	1.320	0.2372	**+0.860**	0.2372	Multivariable	age, gender, race, education level, marital status, poverty–income ratio, smoking status, alcohol.	**Positive**	Extract linear beta for primary pool; OR part → pool C; note…

Note:

Pool	N. Studi	Pooled β	95% CI	I²	τ²	p-value
Pool A	60	+0.310	[+0.255, +0.366]	98.8%	0.024	<0.001
