# Supplementary material for: Epigenetic Age Acceleration as a Modifiable Public Health Target: A Systematic Review and Meta-Analysis of Environmental, Behavioral, and Social Determinants with Development of the MEAB-Index
Source: Int J Mol Sci. 2026 Jun 2;27(11):5032. doi: 10.3390/ijms27115032 (PMC13256709; doi:10.3390/ijms27115032)
Supplement: Supplementary file 1 [file ijms-27-05032-s001.zip › Supplementary Table S2. ΓÇö Pool B.pdf]

Supplementary Table S2. Detailed results of Pool B: standardised beta coefficients or standardised mean differences (SMD) in epigenetic age acceleration (n = 9 studies)

Study ID	First Author	Year	Country	Study Design	Sample Size	Mean Age	Exposure Category	Specific Exposure	Clock Type	EAA Metric	Effect Type	Effect Value	CI Lower	CI Upper	SE	yi	sei	Adjustment Level	Main Covariates	Direction	Notes
1-s2.0-S027249442500372X	Corley	2026	United Kingdom	Longitudinal birth cohort	475	79.1	Gardening	Gardening frequency (never/rarely, sometimes, freq...	—	Telomere length; DNA methylation-based PhenoA.	Standardized beta coefficient; Hazard Ratio	0.142	+0.045	+0.239	0.0490	**+0.142**	0.0490	Multivariable	Age, sex, education, occupational social class, living alone, BMI, smoking status, cardiovascular.	**Positive**	Mixed report: extract standardized beta (pool B) OR HR (pool...
1-s2.0-S030645302500 4275	Rodrigues	2026	United States	Observational, cross-sect...	726	50.4	Aging anxiety	Declining health anxiety; cumulative aging anxiety	GrimAge2; DunedinPACE	Standardized z-scores of epigenetic age accelartion	Standardized beta coefficient (SD)	0.070	+0.010	+0.130	0.0306	**+0.070**	0.0306	Multivariable	Age, race/ethnicity, educational attainment, annual household income, marital status, menopausal satus.	**Positive**	SD-SD standardized; sensitivity pool B
11357_2024_Article_1408	Haoxin Tina Zheng	2025	Australia	prospective cohort with c...	6208	58.8	Physical activity	Physical activity score (log-transformed and stand...	PCPhenoAge, PCGrimAge, bAge, DNAmFitAge, Dune...	Age-adjusted and standardized residuals.	Standardized beta coefficient	-0.070	-0.090	-0.040	0.0128	**-0.070**	0.0128	Multivariable	Age, sex, country of birth, SEIFA score, smoking status, smoking pack-years (log-transformed), alcol	**Negative**	SD-standardized; sensitivity pool B
13148_2024_Article_1637	Willems	2024	Germany; United States	Cross-sectional observati...	1058	42.6	Personality trait	Self-control (Brief Tangney Self-Control Scale in ...	PhenoAge Acceleration; GrimAge Acceleration; ...	Principal-component-based PhenoAge and GrimAg.	Standardized beta coefficient	-0.340	-0.510	-0.170	0.0867	**-0.340**	0.0867	Multivariable	Socioeconomic contexts, BMI, smoking, genetic correlates of low self-control	**Negative**	Standardized beta; sensitivity pool B
DO-HEALTH Bio-Age	Bischoff-Ferrari	2025	Switzerland	RCT	777	75.5	Supplementation and exercise	Vitamin D (2000 IU/day), Omega-3 (1 g/day), home e...	PhenoAge, GrimAge2, DunedinPACE (primary); al...	Standardized change in age acceleration	Standardized mean difference	-0.160	-0.300	-0.020	0.0714	**-0.160**	0.0714	Multivariable	Chronological age, sex, history of falls, BMI, study site, baseline biological age	**Negative**	SMD (Cohen's d equivalent); sensitivity pool B
gbaf194	Li	2025	Australia	cohort	6208	58.8	social relationships	social isolation index; living alone; social activ...	PCPhenoAge; PCGrimAge; bAge; DunedinPACE	age-adjusted standardized residuals	beta coefficient (standardized)	0.030	+0.010	+0.050	0.0102	**+0.030**	0.0102	Multivariable	age, sex, country of birth, socioeconomic status, education level, smoking status, smoking pack-year.	**Positive**	Standardized beta; sensitivity pool B
glad082	Thomas	2023	United States	cross-sectional	42625	47.0	Diet and Physical Activity	Mediterranean diet adherence (MeDi score tertiles)...	PhenoAge (clinical blood chemistry-based algo...	PhenoAge advancement standardized (difference)	standardized beta coefficient (SD)	-0.140	-0.180	-0.110	0.0179	**-0.140**	0.0179	Multivariable	Age, sex, race/ethnicity, total energy intake, NHANES wave, education.	**Negative**	SD units; sensitivity pool B

Supplementary Table S2. Detailed results of Pool B: standardised beta coefficients or standardised mean differences (SMD) in epigenetic age acceleration (n = 9 studies)

Study ID	First Author	Year	Country	Study Design	Sample Size	Mean Age	Exposure Category	Specific Exposure	Clock Type	EAA Metric	Effect Type	Effect Value	CI Lower	CI Upper	SE	yi	sei	Adjustment Level	Main Covariates	Direction	Notes
nutrients-17-01409	Von Holle	2025	United States	cross-sectional analysis ...	1260	56.0	body iron status	serum ferritin; serum iron; transferrin saturation	DNA methylation-based epigenetic clocks (Grim...	epigenetic age acceleration residuals	standardized regression coefficient	0.060	+0.010	+0.110	0.0255	**+0.060**	0.0255	Multivariable	smoking status (current, former, never); education (high school or less, some college/associate	**Positive**	Standardized; sensitivity pool B
s41598-025-25877-6	Chen	2025	United States	Cross-sectional study	23932	49.7	Metabolic biomarker ratio	Uric acid to high-density lipoprotein cholesterol ...	Phenotypic Age (PhenoAge); Klemera–Doubal Met...	Phenotypic Age Acceleration (PhAA); KDM Age	Standardized beta coefficient	1.000	+0.940	1.060	0.0306	**1.000**	0.0306	Multivariable	Age, sex, race, education level, alcohol consumption, smoking status, cardiovascular disease, hypetention	**Positive**	Standardized beta; sensitivity pool B

Note:

Pool	N. Studi	Pooled β	95% CI	I²	τ²	p-value
**Pool B**	9	+0.071	[−0.104, +0.245]	99.3%	0.069	0.427
