# Supplementary material for: Epigenetic Age Acceleration as a Modifiable Public Health Target: A Systematic Review and Meta-Analysis of Environmental, Behavioral, and Social Determinants with Development of the MEAB-Index
Source: Int J Mol Sci. 2026 Jun 2;27(11):5032. doi: 10.3390/ijms27115032 (PMC13256709; doi:10.3390/ijms27115032)
Supplement: Supplementary file 1 [file ijms-27-05032-s001.zip › Supplementary Table S3 ΓÇö Pool C.pdf]

Supplementary Table S3. Detailed results of Pool C: odds ratios for accelerated epigenetic age acceleration (n = 10 studies)

Study ID	First Author	Year	Country	Study Design	Sample Size	Mean Age	Exposure Category	Specific Exposure	Clock Type	EAA Metric	Effect Type	Effect Value (orig.)	CI Lower (orig.)	CI Upper (orig.)	SE	yi (log OR)	sei	OR (back-transf.)	OR CI Lower	OR CI Upper	Adjustment Level	Main Covariates	Direction	Notes
1-s2.0-S0013935125025368	Cristy Stagnar	2026	United Kingdom	case-control	231	—	paternal smoking	father smoking status (smoking vs non-smoking)	Bohlin gestational age clock; Horvath clock; ...	Intrinsic Epigenetic Age Acceleration (IEAA)	odds ratio	2.400	1.160	4.990	0.9770	**+0.875**	0.3722	**2.400**	3.190	146.936	Multivariable	sex stratification; paternal age ≤35 years; maternal non-smoking status; no adjustment for socio-economic	**Positive**	OR; secondary pool C; yi=log(OR)
1-s2.0-S0147651325019074	Hua Fang	2026	United States	cross-sectional	3577	—	environmental toxicants	nicotine metabolites; metals; polyaromatic hydrocal	phenotypic age acceleration	phenotypic age acceleration (phenotypic age)	adjusted odds ratio	2.160	1.350	3.450	0.5357	**+0.770**	0.2394	**2.160**	3.857	31.500	Multivariable	age, gender (male/female), race (Non-Hispanic White/Non-Hispanic Black/Other)	**Positive**	Adjusted OR; secondary pool C; yi=log(OR)
12967_2025_Article_6492	Sheng	2025	United States	cross-sectional	2345	63.5	early-life smoking	age of smoking initiation (ASI) and smoking	DNAm PhenoAge; DunedinPoAm; HorvathTelo	DNAm PhenoAge, DunedinPoAm, HorvathTelo	odds ratio	1.950	1.150	3.290	0.5459	**+0.668**	0.2681	**1.950**	3.158	26.843	Multivariable	age, sex, race, education, BMI, lipid profile, hypertension, diabetes, CKD	**Positive**	OR; secondary pool C; yi=log(OR)
41598_2024_Article_84957	Wen Zhao	2025	Europe	Mendelian randomization	1E+05	—	Sleep traits	Self-reported insomnia; accelerometer-measured	GrimAge; HannumAge; IEAA	Epigenetic age acceleration (EAA)	Odds ratio	1.170	1.040	1.310	0.0689	**+0.157**	0.0589	**1.170**	2.829	3.706	Multivariable	Age, sex, 10 principal components of ancestry, genotyping array, genetic correlation matrix	**Positive**	Logistic regression OR; secondary pool C; yi=log(OR)
cancers-18-00111-v2	Shiqi Hui	2026	United States	cross-sectional	16628	49.0	Dietary inflammation and oxida...	Dietary Inflammatory Index (DII); Dietary Oxidativ	PhenoAge	PhenoAge acceleration	odds ratio	2.190	1.290	3.720	0.6199	**+0.784**	0.2702	**2.190**	3.633	41.264	Multivariable	gender, race, education level, poverty income ratio (PIR), BMI, cotinine, alcohol intake	**Positive**	OR; secondary pool C; yi=log(OR)
Penha_2026_BMC_Medicine	Penha	2026	Australia, Sweden, Ita...	Nested case-control studi...	1480	56.5	Tobacco smoking	Age-adjusted DNA methylation clocks (Hannum)	Chronological age clocks (Hannum), Mortality ...	Age acceleration residuals (age-adjusted DNAm)	Odds ratio per standard deviation.	1.660	1.440	1.910	0.1199	**+0.507**	0.0721	**1.660**	4.221	6.753	Multivariable	Age, sex, cohort, smoking duration, cigarettes per day, years since quitting smoking, BMI	**Positive**	OR per SD; secondary pool C; yi=log(OR)
s12944-025-02446-4	Liu	2025	United States	Cross-sectional analysis ...	2580	38.6	Body fat area; insulin resista...	Visceral fat area (VFA); abdominal subcutaneous.	Phenotypic Age (PhenoAge)	Phenotypic Age Acceleration (PhenoAgeAccel)	Odds Ratio (OR) for PhenoAge Accel	8.339	5.559	12.510	17.732	**2.121**	0.2069	**8.339**	259.563	271.043	Multivariable	Age, gender, race, exercise, alcohol use, smoking status, marital status, education level, income	**Positive**	OR for dichotomized PhenoAgeAccel; secondary pool C; yi=log

Supplementary Table S3. Detailed results of Pool C: odds ratios for accelerated epigenetic age acceleration (n = 10 studies)

Study ID	First Author	Year	Country	Study Design	Sample Size	Mean Age	Exposure Category	Specific Exposure	Clock Type	EAA Metric	Effect Type	Effect Value (orig.)	CI Lower (orig.)	CI Upper (orig.)	SE	yi (log OR)	sei	OR (back-transf.)	OR CI Lower	OR CI Upper	Adjustment Level	Main Covariates	Direction	Notes
s41598-024-84957-1	Wen Zhao	2025	Europe	Mendelian randomization	1E+06	—	Sleep traits	Self-reported insomnia; accelerometer-measured	GrimAge; HannumAge; IEAA	Epigenetic age acceleration	Odds ratio	1.170	1.040	1.310	0.0689	**+0.157**	0.0589	**1.170**	2.829	3.706	Multivariable	Age, sex, 10 principal components of ancestry, genotyping array, genetic correlation matrix	**Positive**	Logistic regression OR; secondary pool C; yi=log(OR)
Wang_2025_Medicine	Wang	2025	United States	cross-sectional	10846	47.3	dietary flavones	total flavones, apigenin, luteolin intake.	Phenotypic age (PhenoAge)	PhenoAgeAccel	odds ratio	0.904	+0.859	+0.953	0.0240	**-0.101**	0.0265	**0.904**	2.361	2.593	Multivariable	age, sex, BMI, ethnicity, poverty status, education, physical activity, marital status	**Negative**	OR; secondary pool C; yi=log(OR)
Yang_2026_PhenoAgeAccel_MetS_C...	Jiaxiang Yang	2026	United States	cross-sectional	12664	—	Metabolic syndrome	Metabolic syndrome (MetS) defined by NCEP ATP III	PhenoAge	PhenoAge acceleration (PhenoAgeAccel)	odds ratio	1.338	1.074	1.666	0.1510	**+0.291**	0.1120	**1.338**	2.927	5.291	Multivariable	age, gender, race/ethnicity, education level, poverty-income ratio, marital status, smoking status	**Positive**	OR; secondary pool C; yi=log(OR)

Note:

Pool	N. Studies	Pooled β	95% CI	I²	τ²	p-value
**Pool C**	10	+0.0559	[−0.299, +0.819]	95.7%	0.144	<0.001
