# Supplementary material for: Epigenetic Age Acceleration as a Modifiable Public Health Target: A Systematic Review and Meta-Analysis of Environmental, Behavioral, and Social Determinants with Development of the MEAB-Index
Source: Int J Mol Sci. 2026 Jun 2;27(11):5032. doi: 10.3390/ijms27115032 (PMC13256709; doi:10.3390/ijms27115032)
Supplement: Supplementary file 1 [file ijms-27-05032-s001.zip › Supplementary Table S4. ΓÇö Pool D.pdf]

Supplementary Table S4. Detailed results of Pool D: hazard ratios for accelerated epigenetic age acceleration (n = 4 studies)

Study ID	First Author	Year	Country	Study Design	Sample Size	Mean Age	Exposure Category	Specific Exposure	Clock Type	EAA Metric	Effect Type	Effect Value (orig.)	CI Lower (orig.)	CI Upper (orig.)	SE	yi (log OR)	sei	OR (back-transf.)	OR CI Lower	OR CI Upper	Adjustment Level	Main Covariates	Direction	Notes
1-s2.0-S0013935125028853	Jilong Bai	2026	United Kingdom	cohort	343723	56.6	residential environment	green space; blue space; natural environment	Phenotypic Age (PhenoAge)	PhenoAge acceleration (residual of PhenoAge)	hazard ratio	0.790	+0.740	+0.850	0.0281	**-0.236**	0.0354	**0.790**	2.096	2.340	Multivariable	age, sex, ethnicity, education, Townsend Deprivation Index,	**Negative**	Cox HR; secondary pool D; yi=log(HR)
1-s2.0-S1279770725002805	Yonghao Li	2026	United Kingdom	cohort	196473	—	dietary patterns	DASH, MEDAS, MIND dietary pattern adherence scores	phenotypic age acceleration	PhenoAgeAccel residuals	hazard ratio	0.750	+0.580	+0.960	0.0969	**-0.288**	0.1285	**0.750**	1.786	2.612	Multivariable	age, sex, BMI, ethnicity, annual household income, education level, smoking status, alcohol consumpt	**Negative**	Cox HR; secondary pool D; yi=log(HR)
Liu_2025_DOM_UK Biobank	Liu	2025	United Kingdom	cohort	54418	56.9	cardiorespiratory fitness	maximal oxygen uptake (VO2max)	biological age (BA) and phenotypic age (Pheno...	age acceleration residuals from regression	hazard ratio	0.720	+0.680	+0.760	0.0204	**-0.329**	0.0284	**0.720**	1.974	2.138	Multivariable	sex, age, ethnicity, smoking status, alcohol status, education level, BMI, overall health	**Negative**	Cox HR; secondary pool D; yi=log(HR)
s12967-025-06189-9	Qiu	2025	United States	cross-sectional	3509	49.7	Inflammatory markers and Helic...	Neutrophil count and systemic inflammatory respons.	Phenotypic age acceleration (PhenoAgeAccel)	PhenoAgeAccel	hazard ratio	1.070	1.060	1.070	0.0026	**+0.068**	0.0024	**1.070**	2.886	2.915	Multivariable	chronological age, sex, race, education, BMI, smoking, drinking behavior, comorbidity (hypertensio).	**Positive**	Cox HR; secondary pool D; yi=log(HR)

Note:

Pool	N. Studi	Pooled	95% CI	I²	τ²	p-value
**Pool D**	4	-0.190	[−0.448 +0.067]	98.9%	0.065	0.146
